# Combination of ultrafast dynamic contrast-enhanced MRI-based radiomics and artificial neural network in assessing BI-RADS 4 breast lesions: Potential to avoid unnecessary biopsies

**DOI:** 10.3389/fonc.2023.1074060

**Published:** 2023-02-01

**Authors:** Yidong Lyu, Yan Chen, Lingsong Meng, Jinxia Guo, Xiangyu Zhan, Zhuo Chen, Wenjun Yan, Yuyan Zhang, Xin Zhao, Yanwu Zhang

**Affiliations:** ^1^ Department I of Breast, Third Affiliated Hospital of Zhengzhou University, Zhengzhou, China; ^2^ Department of Radiology, Third Affiliated Hospital of Zhengzhou University, Zhengzhou, Henan, China; ^3^ General Electric (GE) Healthcare, MR Research China, Beijing, China

**Keywords:** ultrafast dynamic contrast-enhanced MRI, radiomics, neural network, breast imaging reporting and data system, breast cancer

## Abstract

**Objectives:**

To investigate whether combining radiomics extracted from ultrafast dynamic contrast-enhanced MRI (DCE-MRI) with an artificial neural network enables differentiation of MR BI-RADS 4 breast lesions and thereby avoids false-positive biopsies.

**Methods:**

This retrospective study consecutively included patients with MR BI-RADS 4 lesions. The ultrafast imaging was performed using Differential sub-sampling with cartesian ordering (DISCO) technique and the tenth and fifteenth postcontrast DISCO images (DISCO-10 and DISCO-15) were selected for further analysis. An experienced radiologist used freely available software (FAE) to perform radiomics extraction. After principal component analysis (PCA), a multilayer perceptron artificial neural network (ANN) to distinguish between malignant and benign lesions was developed and tested using a random allocation approach. ROC analysis was performed to evaluate the diagnostic performance.

**Results:**

173 patients (mean age 43.1 years, range 18–69 years) with 182 lesions (95 benign, 87 malignant) were included. Three types of independent principal components were obtained from the radiomics based on DISCO-10, DISCO-15, and their combination, respectively. In the testing dataset, ANN models showed excellent diagnostic performance with AUC values of 0.915-0.956. Applying the high-sensitivity cutoffs identified in the training dataset demonstrated the potential to reduce the number of unnecessary biopsies by 63.33%-83.33% at the price of one false-negative diagnosis within the testing dataset.

**Conclusions:**

The ultrafast DCE-MRI radiomics-based machine learning model could classify MR BI-RADS category 4 lesions into benign or malignant, highlighting its potential for future application as a new tool for clinical diagnosis.

## Introduction

Breast cancer is the most common malignant tumor in women and the second leading cause of cancer-related death in women (1). Early cancer detection is beneficial to improve the prognosis of patients with breast cancer (2). Breast magnetic resonance imaging (MRI) plays an important role in the diagnosis (3), treatment (4), and prognostic assessment (5) of breast cancer. American College of Radiology (ACR) Breast Imaging Reporting and Data System (BI-RADS) is helpful for clinical decision-making (6, 7). BI-RADS category 4 lesions with a varying range of probability of malignancy (2%-95%) (6, 8), however, are regarded as suspicious lesions and usually recommended for biopsy (7), which may lead to a large number of negative biopsies (9) as well as the psychological and financial burden for patients. Therefore, it is necessary to find a sensitive tool to improve the assessment of BI-RADS 4 lesions. In order to avoid false positive BI-RADS 4 category assignments, previous studies utilized either advanced MRI techniques (10–13) or specific clinical decision rule incorporated morphological and kinetic BI-RADS descriptors (14, 15). Although these approaches showed encouraging results, additional measurements increase the scan time and the decision rule require human image features interpretation that may lead to inter-reader variation (16).

Radiomics is an emerging field that can non-invasively provide rich information on lesions by quantitatively analyzing numerous features extracted from traditional medical images (17). Different from the conventional visual interpretations of radiologists, this technique can objectively quantify the heterogeneity of diseases. Consequently, radiomics has been successfully explored as a means to aid decision-making for the diagnosis and risk stratification of several kinds of cancers, for example, glioblastoma (18), lung cancer (19), cervical carcinoma (20), and hepatocellular carcinoma (21). Radiomics also shows encouraging results for improving the accuracy of breast cancer diagnosis, prognosis, and prediction of recurrence (22–25).

Ultrafast dynamic contrast-enhanced magnetic resonance imaging (DCE-MRI) is a newly proposed imaging protocol that can provide improved temporal resolution while maintaining reasonable spatial resolution (26, 27). Several fast acquisition techniques have played an important role in ultrafast imaging, consisting of view sharing, sophisticated parallel imaging, and compressed sensing (27–29). Differential sub-sampling with cartesian ordering (DISCO), which utilizes pseudorandom segmentation of the k-space and two-point Dixon fat-water separation, is a type of view-sharing technique (30). All the methods with 3-10 s of temporal resolution can capture kinetic information of a lesion in the very early post-contrast phase. Many studies have demonstrated that the early image features of ultrafast imaging are beneficial for breast cancer diagnosis and characterization (27, 29, 31).

The radiomics models have been developed by some studies to improve the assessment of BI-RADS 4 lesions (32–34). However, they examined the features from conventional sequences including ADC maps, T1W images, T2W images, or T1 contrast-enhanced images. Few studies have reported the diagnostic efficiency of radiomics features based on ultrafast imaging in distinguishing breast suspicious lesions. Therefore, the purpose of this study was (a) to investigate if combining radiomics features extracted from ultrafast imaging (using the DISCO technique) with an artificial neural network (ANN) can differentially diagnose the MR BI-RADS 4 breast lesions, (b) to determine whether and how many false-positive biopsies could be potentially avoided by comparing the results with prospectively prescribed biopsy indications by experienced breast radiologists.

## Materials and methods

### Patients

**Figure 1:**
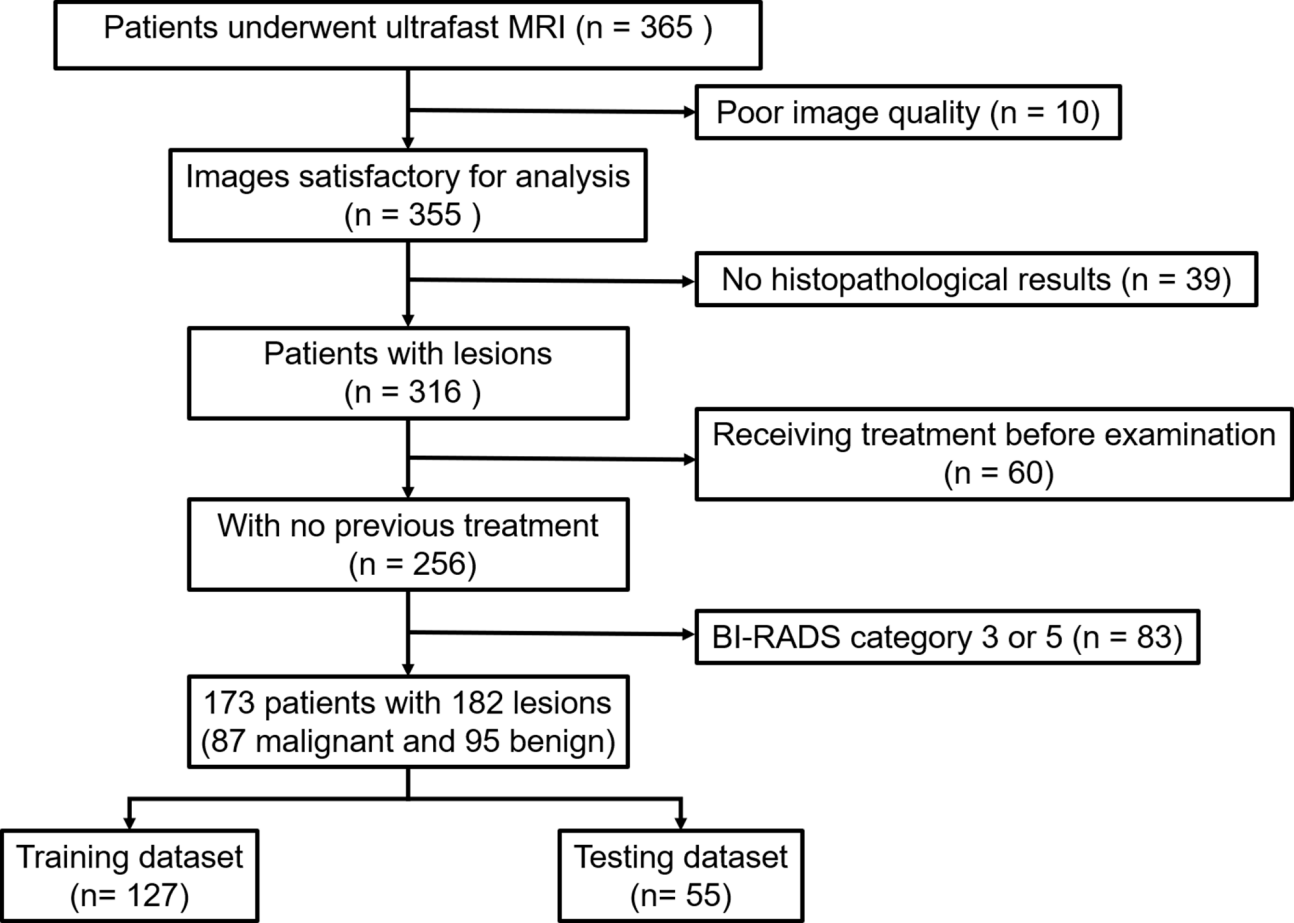
Flowchart of patient selection in this study.

This retrospective study was approved by the Institutional Review Board of our institution and written informed consent was waived. We consecutively reviewed 365 patients who presented suspicious lesions by mammography or breast ultrasonography and underwent breast MRI for diagnosis or preoperative staging from April 2020 to May 2021. Samples of all lesions were obtained by biopsy or surgery and analyzed subsequently by an experienced pathologist. The pathological results of all lesions were regarded as the reference standard. 192 patients were excluded due to the following reasons (1): poor image quality fails to satisfy the diagnostic requirement (n = 10) (2); no pathological results available (n = 39) (3); prior biopsy or chemotherapy before MRI examination (n = 60) (4) BI-RADS category 3 or 5 lesions (n = 83). Finally, 173 patients with 182 MR BI-RADS category 4 lesions were included. Nine patients had breast lesions in both breasts. The flowchart of patient selection is shown in **Figure 1**.

### MRI acquisition protocol

All patients underwent breast MRI in a prone position using a 3.0-T scanner (SIGNA Pioneer, GE Healthcare, Waukesha, WI, USA) with an 8-channel breast coil. The MRI protocol included axial T1-weighted imaging, axial T2-weighted imaging with fat suppression, diffusion-weighted imaging, and dynamic contrast-enhanced (DCE) imaging. DCE-MRI consisted of a pre-contrast image of conventional DCE-MRI, followed by 15 phases of ultrafast DCE-MRI, and then five conventional DCE-MRI. Ultrafast imaging that utilized the DISCO technique was performed with the start of gadolinium-based contrast medium injection. Utilizing an MR power injector, gadolinium diamine (GE Healthcare, Shanghai, China) was administered at a dose of 0.1 mmol/kg of body weight and a rate of 2.5 ml/s, followed immediately by a 20-ml saline flush with the same rate. Only the DISCO images were used for radiomics analysis. The acquisition parameters are shown in **Supplementary Material 1** **Table S1**.

### Image segmentation and feature extraction

The tenth and fifteenth postcontrast DISCO images (hereafter DISCO-10 and DISCO-15, respectively), which were acquired respectively at ~70 and ~105 seconds after contrast was injected, were selected for analysis since the peak contrast time between the lesion and the background peaked was approximately 60-120 seconds (35).

For lesion segmentation, three steps were performed: first, two experienced radiologists (reader 1 with 5 years and reader 2 with 10 years of experience in reading breast images, respectively) reviewed the images in consensus identifying the location of the targeted lesion before sketching the region of interest (ROI). Both readers were blinded to initial radiological reports and the pathologic outcomes. Second, reader 1 manually sketched the ROI by using ITK-SNAP software (version 3.6.0, www.itksnap.org). The ROI traced the borders of each lesion and included the entire enhancing area. This step yielded a 3-dimensional (3D) ROI. Third, all segmentations were reviewed by a senior radiologist (reader 3 with more than 15 years of experience) and revised as necessary by adding or replacing seed points.

To evaluate the intra- and interobserver consistency of image segmentation and feature, 50 cases were randomly selected. Reader 1 repeatedly draw the ROIs four weeks later. Reader 3 (with more than experience of 15 years) who was blinded to pathological information independently outlined the ROIs according to the same procedure. The intraclass correlation coefficient (ICC) was used to evaluate intra- and interobserver agreement and ICC > 0.75 was regarded as a satisfactory result (20, 36).

In this study, radiomics extraction was performed by using a freely available software named FeAture Explorer version 5.0 (FAE 5.0) (37). A total of 107 features were automatically extracted from each lesion ROI, consisting of 18 histogram features, 14 shape features, and 75 texture features. The texture features included gray level co-occurrence matrix (GLCM) (24 features), gray-level dependence matrix (GLDM) (14 features), gray level run length matrix (GLRLM) (16 features), gray level size zone matrix (GLSZM) (16 features), neighboring gray-tone difference matrix (NGTDM) (5 features). The details of the features are summarized in **Supplementary Material 1** **Table S2**.

### Feature selection and model development

**Figure 2:**
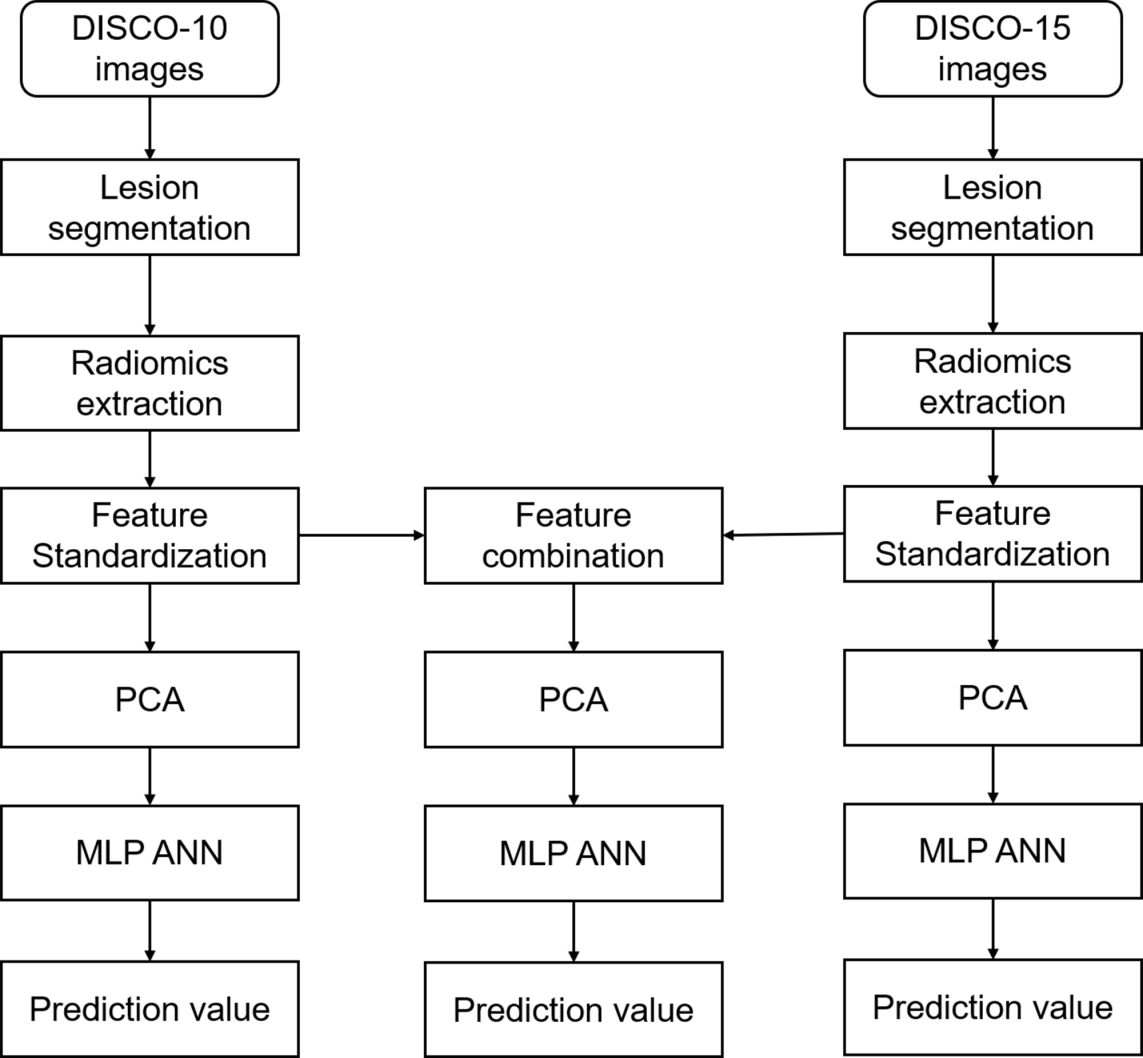
Flowchart of radiomics analysis in this study. *DISCO*, Differential sub-sampling with cartesian ordering; *PCA*, principal component analysis; *MLP ANN*, multilayer perceptron artificial neural network.

For feature selection, we performed the principal component analysis (PCA) with varimax rotation using SPSS software (version 26.0, IBM) and utilized Kaiser’s criterion (eigenvalue > 1) to select the components for further analysis. Three different kinds of principal components were obtained from the features of DISCO-10, DISCO-15, and their combination, respectively (**Figure 2**).

A multilayer perceptron (MLP) artificial neural network (ANN) was performed to develop the predictive models using the selected components as input. The output layer of the model was the likelihood of malignancy using histological results as the gold standard. The ANN architecture was determined using an automatic selection method based on optimal diagnostic efficiency. The number of units in the hidden layer was set between 1 and 50. To improve inter-reader comparability, we set a seed of 20220928 as a random number generator. The training was performed in batch mode utilizing the scaled conjugate gradient as an optimization algorithm. The initial lambda was set to 0.0000005, sigma to 0.00005, interval center to 0, and interval offset to 0.5. For stopping rules, the maximum steps without a decrease in an error of 1, the minimum relative change in training error of 0.0001, and the minimum relative change in training error ratio of 0.001 were adopted. The data of computing prediction error and the number of training epochs were automatically chosen. The dataset was randomly split into a training set and a testing set in a ratio of approximately 7:3. All radiomics models were trained based on the training set, and then tested based on the testing set. All analyses were performed using SPSS software (version 26.0, IBM).

### Statistical analysis

The receiver operating characteristic (ROC) curve analysis was performed to assess the diagnostic performance using histopathology as the reference standard. The area under the curve (AUC), sensitivity, specificity, positive predictive value (PPV), and negative predictive value (NPV) were calculated. The exploratory cutoff value was selected within the training dataset with a sensitivity of approximately 90% or above and validated using the testing dataset. When the AUC in the training dataset is significantly higher than in the testing dataset, the model is assumed to be overfitted. The differences in AUCs and specificity for different models were compared using the Delong test and McNemar test, respectively. All statistical analyses were performed using the statistical software SPSS version 26.0 (IBM) and MedCalc version 19.8 (MedCalc). *P* < 0.05 was considered statistically significant.

## Results

### Population and lesion descriptors

A total of 173 patients (mean age, 43.1 ± 11.7 years; range 18-69 years); with 182 lesions were included in the study. Histopathology identified 95 (52.2%) benign and 87 (47.8%) malignant lesions, including invasive ductal carcinoma 68 (78.2%), ductal carcinoma *in situ* 14 (16.1%), mucinous carcinoma 4 (4.6%), invasive lobular carcinoma 1 (1.1%). The mean lesion size was 2.3 ± 1.6 cm. After the random split, approximately 70% (127/182) of cases were regarded as the training dataset and 30% (55/182) as the testing dataset. The detailed results are summarized in **Table 1**.

**Table 1:** Histopathology results in this study.

Histology	Training (n = 127)	Testing (n= 55)
Malignant	62 (48.8%)	25 (45.5%)
Invasive ductal carcinoma	47 (75.8%)	21 (84%)
Ductal carcinoma in situ	10 (16.1%)	4 (16%)
Mucinous carcinoma	4 (6.5%)	
Invasive lobular carcinoma	1 (1.6%)	
Benign	65 (51.2%)	30 (54.5%)
Fibroadenomas	34 (52.3%)	19 (63.4%)
Adenosis	15 (23.1%)	8 (26.7)
Papilloma	9 (13.9%)	1 (3.3)
Inflammation	6 (9.2%)	1 (3.3)
Phyllodes tumor	1 (1.5%)	1 (3.3)

### PCA of radiomic features

We obtained excellent intra- and inter-observer consistency in 214 (107 × 2) features and no feature was removed. The mean ICCs were 0.96 (*P* < 0.001) and 0.92 (*P* < 0.001) for intra- and inter-observer, respectively.

PCA was performed and yielded three categories of principal components (PC), consisting of eleven PC for DISCO-10, eleven PC for DISCO-15, and sixteen PC for their combination, respectively. The PC was utilized as input layers for the multilayer perceptron ANN. The detailed results of the PCA are summarized in **Supplementary Materials 2–4**.

### Diagnostic performance of ANN

**Figure 3:**
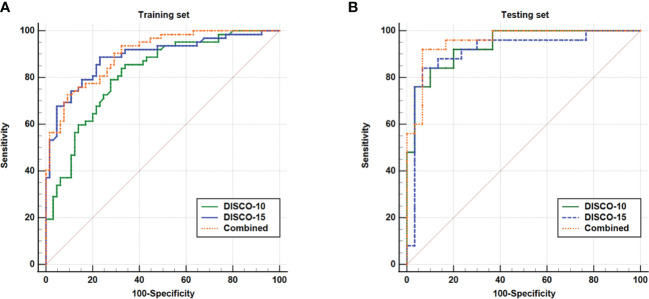
ROC curves of the ANN for the training **(A)** and testing **(B)** datasets.

**Figure 3** illustrates the ROC curves of different models within the training and testing dataset. The AUC of DISCO-10, DISCO-15, and their combination was 0.817 (95%CI, 0.739-0.880), 0.889 (95%CI, 0.821-0.938), and 0.902 (95%CI, 0.836-0.948) in training dataset and 0.937 (95%CI, 0.838-0.985), 0.915 (95%CI, 0.808-0.973), and 0.956 (95%CI, 0.864-0.993) in the testing dataset, respectively (**Table 2**). Compared with the training dataset, the AUC values in the testing dataset were higher for DISCO-10 (*P* = 0.012), DISCO-15 (*P* = 0.625), and the combined method (*P* = 0.127), which indicated that classification models were not overfitted. On the testing dataset, the combined scheme yielded the highest AUC value compared with the single sequence radiomics model based on DISCO-10 (*P* = 0.294), and DISCO-15 (*P* = 0.122). DISCO-10 achieved a slightly higher AUC in comparison with DISCO-15 (*P* = 0.411). The details of the ANN architecture are provided in **Supplementary Materials 5**.

**Table 2:** Comparison for AUCs of different models within the training and testing set.

	AUC	SE	95%CI
Training (n = 127)			
DISCO-10	0.817	0.037	0.739 - 0.880
DISCO-15	0.889	0.030	0.821 - 0.938
Combined	0.902	0.025	0.836 - 0.948
Testing (n = 55)			
DISCO-10	0.937	0.030	0.838 - 0.985
DISCO-15	0.915	0.044	0.808 - 0.973
Combined	0.956	0.025	0.864 - 0.993

Pairwise comparison of ROC curves within the testing set: Combined vs. DISCO-10 (P = 0.294); Combined vs. DISCO-15 (P = 0.122); DISCO-10 vs. DISCO-15 (P = 0.411).

AUC, area under the curve; SE, standard error; CI, confidence interval.

### Potential of the ANN to avoid unnecessary biopsies

**Figure 4:**
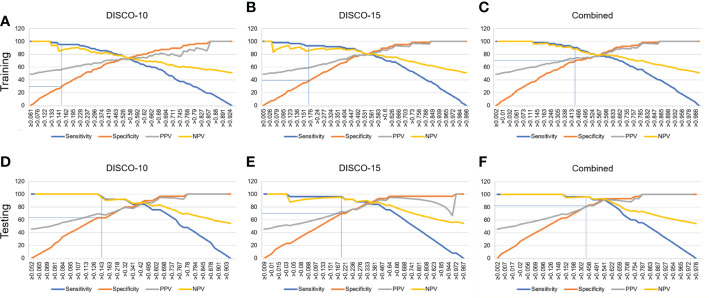
Estimated proportion of sensitivity, specificity, PPV, and NPV (y-axis) at different predicted probability thresholds (x-axis) for training dataset **(A-C)** and testing dataset **(D-F)**. **(A-C)** The vertical blue lines indicate the cutoff values of 0.144, 0.171, and 0.459 at high sensitivity (>90%) for DISCO-10, DISCO-15, and combined methods within the training dataset, respectively. **(D-F)** The vertical blue lines indicate the diagnostic performance within the testing dataset using the predefined cutoff values. PPV, positive predictive value; NPV, negative predictive value.

In this study, three exploratory cut-off values (> 0.144, > 0.171, > 0.459) predicted probability of malignancy were identified in the training dataset, yielding the sensitivity of 95.16%, 93.55%, 90.32%, and the specificity of 20.69%, 38.46%, and 70.77%, respectively (**Table 3**, **Figure 4**).

**Table 3:** Diagnostic performance of the ANN models.

	Criterion	Sensitivity (%) (TP/TP + FN)	95% CI	Specificity (%) (TN/TN + FP)	95% CI	PPV	NPV
Training (n = 127)							
DISCO-10	>0.144	95.16 (59/62)	86.5-99.0	27.69 (18/65)	17.3-40.2	55.7	85.7
DISCO-15	>0.171	93.55 (58/62)	84.3-98.2	38.46 (25/65)	26.7-51.4	59.2	86.2
Combined	>0.459	90.32 (56/62)	80.1-96.4	70.77 (46/65)	58.2-81.4	74.7	88.5
Testing (n = 55)							
DISCO-10	>0.144	96.00 (24/25)	79.6-99.9	63.33 (19/30)	43.9-80.1	68.6	95.0
DISCO-15	>0.171	96.00 (24/25)	79.6-99.9	70.00 (21/30)	50.6-85.3	72.7	95.5
Combined	>0.459	96.00 (24/25)	79.6-99.9	83.33 (25/30)	65.3-94.4	82.8	96.2

Comparison of specificity for different models within the testing set: Combined vs. DISCO-10 (P = 0.109); Combined vs. DISCO-15 (P = 0.289); DISCO-10 vs. DISCO-15 (P = 0.727).

TP, true positive; FN, false negative; TN, true negative; FP, false positive; CI, confidence interval; PPV, positive predictive value; NPV, negative predictive value; DISCO, Differential sub-sampling with cartesian ordering.

**Figure 5:**
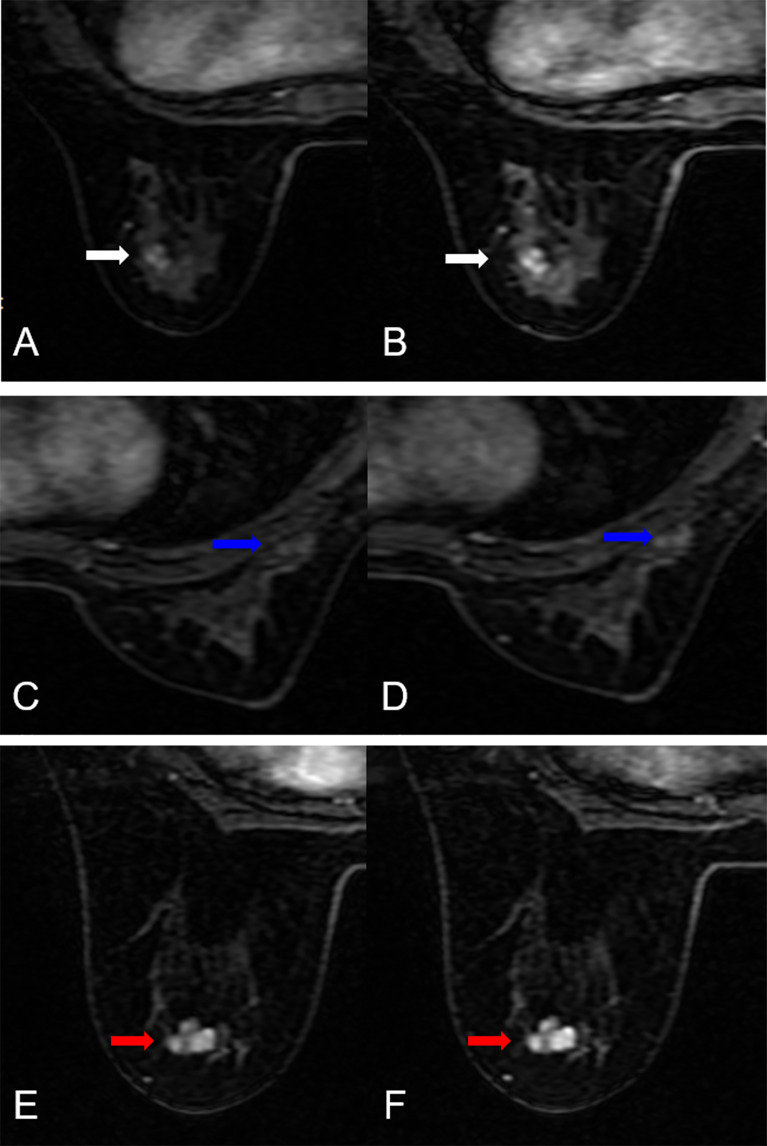
False-negative and false-positive results. **(A, B)** False-negative case: A 51-year-old female patient: MRI showed an irregular lesion in the left breast (white arrow. The lesion demonstrated heterogeneous internal enhancement **(A, B)**. **(A)** DISCO-10, **(B)** DISCO-15. The ANN classifiers predicted a low likelihood of malignancy (14.3% for DISCO-10, 4.2% for DISCO-15, and 15.2% for combined, respectively). Histology revealed an invasive ductal carcinoma. **(C–F)** False-positive cases. **(C, D)** A 34-year-old female patient: MRI showed an irregular lesion in the right breast (blue arrow). The lesion demonstrated heterogeneous internal enhancement **(C, D)**. **(C)** DISCO-10, **(D)** DISCO-15. The ANN classifiers predicted a high likelihood of malignancy (65.6% for DISCO-10, 64.0% for DISCO-15, and 75.4% for combined, respectively). Histology revealed an adenosis. **(E, F)** A 35-year-old female patient: MRI showed an irregular lesion in the left breast (red arrow). The lesion demonstrated heterogeneous internal enhancement **(E, F)**. **(E)** DISCO-10, **(F)** DISCO-15. The ANN classifiers predicted a high likelihood of malignancy (78.0% for DISCO-10, 97.2% for DISCO-15, and 78.7% for combined, respectively). Histology revealed a fibroadenoma.

In the testing dataset, evaluating the diagnostic performance of the DISCO-10 using the predefined cut-off value (> 0.144) showed a sensitivity of 96% and a specificity of 63.33%. For the diagnostic performance of DISCO-15, applying the cut-off value (> 0.171) resulted in a sensitivity of 96% and a specificity of 70%. When using the exploratory cut-off value of 0.459 of the combined method, the sensitivity and specificity were 96% and 83.33%, respectively (**Table 3**, **Figure 4**). By means of three ANN models, nineteen of 30, twenty-one of 30, and twenty-five of 30 benign breast lesions were correctly diagnosed, while leading to one false-negative diagnosis respectively (**Table 3**). The combined scheme showed slightly higher specificity compared with DISCO-10 (*P* = 0.109) and DISCO-15 (*P* = 0.289), but not significantly. The false-negative and false-positive diagnoses using different ANN models within the testing set at a sensitivity of 96% are summarized in **Table 4**. Representative clinical cases are illustrated in **Figure 5**.

**Table 4:** False-negative and false-positive diagnoses using different models within the testing set at high level of sensitivity (96%).

	False negatives	n	False positives	n
DISCO-10		1		11
	Invasive ductal carcinoma	1	Fibroadenoma	7
			Adenosis	3
			Inflammation	1
DISCO-15		1		9
	Invasive ductal carcinoma	1	Fibroadenoma	8
			Adenosis	1
Combined		1		5
	Invasive ductal carcinoma	1	Fibroadenoma	4
			Adenosis	1

## Discussion

We demonstrated that the investigated ultrafast DCE-MRI-based radiomics combined with ANN could be used to differentially diagnose the MR BI-RADS 4 lesions. The constructed classifiers showed good discriminative performance with the AUC values ranging from 0.915 to 0.956. Rather than assigning a category that was only associated with a variable range of malignant tumor rates, the MLP classifier provided individually predicted likelihood of malignancy. Applying the high-sensitivity cutoffs for breast cancer might have avoided 63.33%-83.33% of all unnecessary biopsies at the price of one false-negative diagnosis.

Although MR BI-RADS category 4 lesions show varying malignancy rates, biopsies are usually recommended in clinical practice, resulting in a substantial number of false positive lesions and a waste of medical resources. Therefore, methods for improving pre-interventional lesion assessment are warranted. Radiomics is increasingly considered an important diagnostic tool, providing quantitative multi-dimensional features extracted from imaging data that may reflect the potential phenotype of tumor disease (17). Many studies have shown that radiomics is useful in evaluating MR BI-RADS 4 lesions. Hu et al. (32) developed a radiomics nomogram based on an apparent diffusion coefficient map to differentially diagnose BI-RADS 4 findings and found a moderate diagnostic performance with an AUC of 0.79, which was lower compared to our results. The possible reason may be that the ultrafast DCE series could provide more information in differentiating breast lesions compared with ADC (38). Zhang et al. (34) and Cui et al. (33) applied MRI-based radiomics models to predict the benignity and malignancy of BI-RADS 4 lesions and yielded a good diagnostic efficiency with the AUC of 0.939 and 0.94, respectively, which were comparable to our results. While in this study, the radiomics were extracted from ultrafast DCE-MRI, which appeared to reduce greatly magnet time.

Avoiding unnecessary biopsies remains a hot topic in the clinical management of breast lesions. Currently, a clinical decision rule named the Kaiser score has been proposed to assess breast lesions, with improved diagnostic accuracy and the potential to avoid unnecessary biopsies (14, 39–43). Although this method may simplify the image interpretation compared with BI-RADS assignment, differences resulting from experience at different levels (44) and inter-observer variation (16) remain. Interestingly, our results showed that there was no significant difference in the diagnostic performance between the radiomics models and the Kaiser score (**Supplementary Materials 6**). This indicated that the radiomics-based machine learning model might provide comparable results compared with the Kaiser score method while not required to perform image feature interpretation.

The exploratory cutoff at high sensitivity may be used to evaluate the number of avoidable false-positive biopsies (15, 16, 45). Utilizing the radiomics derived from ultrafast DCE-MRI combined with the MLP ANN classifier, we identified that about 63.33%-83.33% of unnecessary biopsies might have been avoided in the testing dataset while maintaining a high sensitivity (96%, 24/25). This was an encouraging result, which had the potential to provide more valuable information to support clinical decision-making.

Currently, abbreviated breast MRI, which can substantially shorten examination and reading times, has been proposed for increasing access to screening for women at average risk of breast cancer. Many studies have demonstrated that abbreviated MRI can improve cancer detection in women with dense breasts (46, 47). However, this protocol may result in unnecessary biopsies because of the lack of kinetics information provided by DCE-MRI. Ultrafast imaging can fill this gap. The features including maximum slope and time to enhancement derived from ultrafast sequences have shown important value in improving tumor characterization, identifying prognostic factors, and assessing treatment (29, 31, 48, 49). But the calculation of these parameters requires commercial software that may not be universally available. In the present study, we explored a more intuitive and easy-to-apply method in a representative patient population and analyzed its clinical utility. To make the proposed approach available to every physician, open-source software was used to perform radiomics extraction from the initial DICOM images. Exporting the learned weights of a well-trained ANN classifier into excel would allow our findings to be quickly integrated into clinical workflows and make it easy to obtain personal predictions of the malignant rates in patients with MR BI-RADS category 4 lesions. However, it should be noted that this is only a preliminary study in the investigated setting and larger patient cohorts are required to validate the results.

While DISCO-15 might have avoided more unnecessary biopsies compared with DISCO-10, not significantly. And the former showed a higher AUC value. In addition, combining DISCO-15 features with DISCO-10 did not yield significantly improved AUC and specificity. This might suggest that the late postcontrast phase of ultrafast DCE-MRI could provide little information for significantly improving diagnostic performance.

There are several limitations in this study. First, the number of cases was relatively small, and it was a retrospective study merely performed in our institution. In order to validate the robustness of the classifier, larger datasets from multicenter are needed in further research. Second, only ultrafast imaging was used to perform radiomics analysis, other sequences, such as DWI, T2WI, and high-spatial-resolution DCE sequences were not included and required further investigation. Third, we analyzed the features of BI-RADS 4 lesions, and other category lesions consisting of category 3 or 5 were not included. We believe that the proposed ANN classifier might also be beneficial in evaluating these lesions and we are currently examining this assumption (4). In this study, the risk factors such as patient age, personal disease history, and gene mutation that are highly associated with breast cancer were not included. The fusion of these features with principal component analysis might yield more stable and trustable diagnostic performance.

In conclusion, our preliminary results indicated that radiomics extracted from ultrafast DCE-MRI imaging combined with the multilayer perceptron artificial neural network could differentially diagnose MR BI-RADS category 4 breast lesions with excellent diagnostic performance, and have the potential to avoid more than 63.33% of unnecessary biopsies. Further investigation with larger patient cohorts is warranted to validate our results in the future.

## Data availability statement

The datasets presented in this article are not readily available because of institutional restrictions. Requests to access the datasets should be directed to Xin Zhao, zhaoxinxct@vip.163.com.

## Ethics statement

The studies involving human participants were reviewed and approved by the Third Affiliated Hospital of Zhengzhou University. Written informed consent for participation was not required for this study in accordance with the national legislation and the institutional requirements. Written informed consent was not obtained from the individual(s) for the publication of any potentially identifiable images or data included in this article.

## Author contributions

Conception and design, YL. Administrative support, XZ and YWZ. Provision of study materials or patients, YC, XYZ, and ZC. Collection and assembly of data, WY, YYZ, and YC. Data analysis and interpretation, YL, LM, and JG. Writing - review and editing, YL, LM, and JG. Final approval of manuscript, all authors. All authors contributed to the article and approved the submitted version.
