# Supplementary material for: Combination of ultrafast dynamic contrast-enhanced MRI-based radiomics and artificial neural network in assessing BI-RADS 4 breast lesions: Potential to avoid unnecessary biopsies
Source: Front Oncol. 2023 Feb 1;13:1074060. doi: 10.3389/fonc.2023.1074060 (PMC9929366; doi:10.3389/fonc.2023.1074060)
Supplement: Supplementary file 1 [file Table_1.docx]

**Supplementary material 1**

**Table S1** MRI sequence parameters

|  | T1WI | T2WI | DWI | DISCO | Conventional DCE |
| --- | --- | --- | --- | --- | --- |
| Scan plane | Axial | Axial | Axial | Axial | Axial |
| TR/TE, (msec) | 813/Min | 7281/77 | 3000/72 | 4.8/Min | 4.4/2 |
| Flip angle, (°) | 111 | 111 | 90 | 15 | 15 |
| NEX | 1 | 1.5 | 5 | 1 | 1 |
| Acquisition matrix | 352×224 | 512×512 | 132×132 | 288×160 | 320×320 |
| Field of view, (cm^2^) | 32×32 | 32×32 | 32×32 | 32×32 | 32×32 |
| Slice thickness, (mm) | 5 | 5 | 5 | 1.2 | 1.2 |
| Slice gap, (mm) | 1 | 1 | 1 | 0 | 0 |
| Number of slices | 32 | 32 | 32 | 134 | 134 |
| Fat suppression | OFF | ON | ON | ON | ON |
| b-value, (s/mm^2^) | - | - | 0/800 | - | - |
| Time per phase, (sec) | - | - | - | 7 | 1:14 |
| Number of phases | - | - | - | 15 | 5 |
| Acquisition time, (min) | 00:43 | 3:17 | 2:42 | 1:45 | 6:10 |

*TR/TE,* repetition time/echo time; *NEX,* number of excitations; *DISCO,* Differential Subsampling with Cartesian Ordering; *T1WI,* T1 weighted imaging; *T2WI,* T2 weighted imaging; *DWI,* diffusion weighted imaging; *DCE,* dynamic contrast-enhanced

**Radiomics features**

A total of 107 features were automatically extracted from each lesion ROI, consisting of 18 histogram features, 14 shape features, and 75 texture features. The texture features included gray level co-occurrence matrix (GLCM) (24 features), gray-level dependence matrix (GLDM) (14 features), gray level run length matrix (GLRLM) (16 features), gray level size zone matrix (GLSZM) (16 features), neighboring gray-tone difference matrix (NGTDM) (5 features). The detailed information is showed in **Table S2**.

**Table S2** Radiomics extracted from each ROI

| **Histogram features (n = 18)** | **Shape features (n = 14)** | **Texture features (n = 75)** | | | | |
| --- | --- | --- | --- | --- | --- | --- |
|  |  | GLCM (n = 24) | GLDM (n = 14) | GLRLM (n = 16) | GLSZM (n = 16) | NGTDM (n = 5) |
| 10Percentile | Elongation | Autocorrelation | DependenceEntropy | GrayLevelNonUniformity | GrayLevelNonUniformity | Busyness |
| 90Percentile | Flatness | ClusterProminence | DependenceNonUniformity | GrayLevelNonUniformityN | GrayLevelNonUniformityN | Coarseness |
| Energy | LeastAxisLength | ClusterShade | DependenceNonUniformityN | GrayLevelVariance | GrayLevelVariance | Complexity |
| Entropy | MajorAxisLength | ClusterTendency | DependenceVariance | HighGrayLevelRunEmphasi | HighGrayLevelZoneEmphas | Contrast |
| InterquartileRange | Maximum2DDiameterColumn | Contrast | GrayLevelNonUniformity | LongRunEmphasis | LargeAreaEmphasis | Strength |
| Kurtosis | Maximum2DDiameterRow | Correlation | GrayLevelVariance | LongRunHighGrayLevelEmp | LargeAreaHighGrayLevelE |  |
| Maximum | Maximum2DDiameterSlice | DifferenceAverage | HighGrayLevelEmphasis | LongRunLowGrayLevelEmph | LargeAreaLowGrayLevelEm |  |
| Mean | Maximum3DDiameter | DifferenceEntropy | LargeDependenceEmphasis | LowGrayLevelRunEmphasis | LowGrayLevelZoneEmphasi |  |
| MeanAbsoluteDeviat | MeshVolume | DifferenceVariance | LargeDependenceHighGrayL | RunEntropy | SizeZoneNonUniformity |  |
| Median | MinorAxisLength | Id | LargeDependenceLowGrayLe | RunLengthNonUniformity | SizeZoneNonUniformityNo |  |
| Minimum | Sphericity | Idm | LowGrayLevelEmphasis | RunLengthNonUniformityN | SmallAreaEmphasis |  |
| Range | SurfaceArea | Idmn | SmallDependenceEmphasis | RunPercentage | SmallAreaHighGrayLevelE |  |
| RobustMeanAbsolute | SurfaceVolumeRatio | Idn | SmallDependenceHighGrayL | RunVariance | SmallAreaLowGrayLevelEm |  |
| RootMeanSquared | VoxelVolume | Imc1 | SmallDependenceLowGrayLe | ShortRunEmphasis | ZoneEntropy |  |
| Skewness |  | Imc2 |  | ShortRunHighGrayLevelEm | ZonePercentage |  |
| TotalEnergy |  | InverseVariance |  | ShortRunLowGrayLevelEmp | ZoneVariance |  |
| Uniformity |  | JointAverage |  |  |  |  |
| Variance |  | JointEnergy |  |  |  |  |
|  |  | JointEntropy |  |  |  |  |
|  |  | MCC |  |  |  |  |
|  |  | MaximumProbability |  |  |  |  |
|  |  | SumAverage |  |  |  |  |
|  |  | SumEntropy |  |  |  |  |
|  |  | SumSquares |  |  |  |  |
