# Supplementary material for: Combination of ultrafast dynamic contrast-enhanced MRI-based radiomics and artificial neural network in assessing BI-RADS 4 breast lesions: Potential to avoid unnecessary biopsies
Source: Front Oncol. 2023 Feb 1;13:1074060. doi: 10.3389/fonc.2023.1074060 (PMC9929366; doi:10.3389/fonc.2023.1074060)
Supplement: Supplementary file 2 [file Table_2.doc]

Supplementary materials 2

PCA analysis of the radiomics based on DISCO-10

Total Variance Explained	
Component	Initial Eigenvalues	Extraction Sums of Squared Loadings	Rotation Sums of Squared Loadings	
	Total	% of Variance	Cumulative %	Total	% of Variance	Cumulative %	Total	% of Variance	Cumulative %	
1	48.591	45.412	45.412	48.591	45.412	45.412	32.427	30.306	30.306	
2	21.373	19.975	65.387	21.373	19.975	65.387	22.368	20.905	51.211	
3	8.505	7.948	73.336	8.505	7.948	73.336	16.713	15.620	66.830	
4	7.029	6.569	79.905	7.029	6.569	79.905	11.051	10.328	77.159	
5	4.221	3.945	83.850	4.221	3.945	83.850	4.306	4.024	81.183	
6	3.182	2.974	86.824	3.182	2.974	86.824	3.122	2.917	84.100	
7	2.522	2.357	89.181	2.522	2.357	89.181	2.936	2.744	86.844	
8	1.912	1.787	90.968	1.912	1.787	90.968	2.678	2.503	89.347	
9	1.778	1.662	92.630	1.778	1.662	92.630	2.643	2.470	91.817	
10	1.328	1.241	93.871	1.328	1.241	93.871	1.888	1.764	93.581	
11	1.119	1.046	94.917	1.119	1.046	94.917	1.429	1.335	94.917	
12	.877	.819	95.736							
13	.712	.665	96.401							
14	.599	.560	96.961							
15	.593	.554	97.515							
16	.382	.357	97.871							
17	.325	.303	98.175							
18	.275	.257	98.432							
19	.230	.215	98.647							
20	.208	.195	98.841							
21	.167	.156	98.998							
22	.149	.139	99.137							
23	.141	.132	99.269							
24	.114	.107	99.375							
25	.100	.094	99.469							
26	.070	.065	99.535							
27	.060	.056	99.591							
28	.055	.052	99.642							
29	.043	.040	99.683							
30	.039	.036	99.719							
31	.039	.036	99.755							
32	.030	.028	99.783							
33	.028	.026	99.809							
34	.024	.022	99.831							
35	.022	.021	99.852							
36	.019	.018	99.870							
37	.017	.016	99.886							
38	.014	.013	99.899							
39	.013	.012	99.911							
40	.010	.009	99.920							
41	.009	.009	99.929							
42	.008	.008	99.936							
43	.008	.007	99.943							
44	.006	.006	99.949							
45	.006	.006	99.955							
46	.006	.006	99.960							
47	.005	.005	99.965							
48	.004	.004	99.969							
49	.004	.004	99.973							
50	.004	.003	99.976							
51	.003	.003	99.979							
52	.003	.003	99.982							
53	.003	.002	99.984							
54	.002	.002	99.986							
55	.002	.002	99.988							
56	.002	.002	99.989							
57	.001	.001	99.991							
58	.001	.001	99.992							
59	.001	.001	99.993							
60	.001	.001	99.994							
61	.001	.001	99.995							
62	.001	.001	99.996							
63	.001	.001	99.997							
64	.001	.001	99.997							
65	.000	.000	99.997							
66	.000	.000	99.998							
67	.000	.000	99.998							
68	.000	.000	99.999							
69	.000	.000	99.999							
70	.000	.000	99.999							
71	.000	.000	99.999							
72	.000	.000	99.999							
73	.000	9.540E-5	99.999							
74	9.435E-5	8.817E-5	100.000							
75	8.658E-5	8.092E-5	100.000							
76	7.374E-5	6.891E-5	100.000							
77	6.087E-5	5.689E-5	100.000							
78	5.432E-5	5.077E-5	100.000							
79	4.378E-5	4.091E-5	100.000							
80	3.528E-5	3.297E-5	100.000							
81	2.860E-5	2.673E-5	100.000							
82	2.749E-5	2.569E-5	100.000							
83	2.289E-5	2.139E-5	100.000							
84	1.517E-5	1.418E-5	100.000							
85	1.455E-5	1.360E-5	100.000							
86	1.094E-5	1.022E-5	100.000							
87	1.021E-5	9.542E-6	100.000							
88	8.045E-6	7.518E-6	100.000							
89	6.677E-6	6.240E-6	100.000							
90	6.193E-6	5.788E-6	100.000							
91	4.112E-6	3.843E-6	100.000							
92	3.965E-6	3.705E-6	100.000							
93	2.870E-6	2.683E-6	100.000							
94	1.418E-6	1.325E-6	100.000							
95	8.243E-7	7.703E-7	100.000							
96	7.698E-7	7.194E-7	100.000							
97	4.836E-7	4.519E-7	100.000							
98	2.357E-7	2.202E-7	100.000							
99	7.864E-8	7.349E-8	100.000							
100	5.781E-8	5.403E-8	100.000							
101	3.609E-8	3.373E-8	100.000							
102	1.419E-8	1.326E-8	100.000							
103	6.016E-9	5.623E-9	100.000							
104	7.910E-10	7.392E-10	100.000							
105	7.722E-16	7.217E-16	100.000							
106	1.034E-16	9.663E-17	100.000							
107	-4.727E-16	-4.418E-16	100.000							

Extraction Method: Principal Component Analysis.	


Rotated Component Matrixa	
	Component	
	1	2	3	4	5	6	7	8	9	10	11	
ZWATER__Ph10_Ax_3D_DISCO_C_original_glszm_GrayLevelVariance	.962											
ZWATER__Ph10_Ax_3D_DISCO_C_original_glrlm_GrayLevelVariance	.959											
ZWATER__Ph10_Ax_3D_DISCO_C_original_firstorder_Variance	.959											
ZWATER__Ph10_Ax_3D_DISCO_C_original_gldm_GrayLevelVariance	.959											
ZWATER__Ph10_Ax_3D_DISCO_C_original_glcm_SumSquares	.951											
ZWATER__Ph10_Ax_3D_DISCO_C_original_glcm_ClusterTendency	.946											
ZWATER__Ph10_Ax_3D_DISCO_C_original_gldm_SmallDependenceHighGray	.945											
ZWATER__Ph10_Ax_3D_DISCO_C_original_glszm_SmallAreaHighGrayLevel	.929											
ZWATER__Ph10_Ax_3D_DISCO_C_original_glcm_Autocorrelation	.911											
ZWATER__Ph10_Ax_3D_DISCO_C_original_glszm_HighGrayLevelZoneEmpha	.909											
ZWATER__Ph10_Ax_3D_DISCO_C_original_glrlm_ShortRunHighGrayLevelE	.908											
ZWATER__Ph10_Ax_3D_DISCO_C_original_glrlm_HighGrayLevelRunEmphas	.905											
ZWATER__Ph10_Ax_3D_DISCO_C_original_gldm_HighGrayLevelEmphasis	.904											
ZWATER__Ph10_Ax_3D_DISCO_C_original_ngtdm_Complexity	.904											
ZWATER__Ph10_Ax_3D_DISCO_C_original_glcm_DifferenceVariance	.901											
ZWATER__Ph10_Ax_3D_DISCO_C_original_firstorder_MeanAbsoluteDevia	.892	.403										
ZWATER__Ph10_Ax_3D_DISCO_C_original_glrlm_LongRunHighGrayLevelEm	.890											
ZWATER__Ph10_Ax_3D_DISCO_C_original_firstorder_RobustMeanAbsolut	.887											
ZWATER__Ph10_Ax_3D_DISCO_C_original_firstorder_InterquartileRang	.884											
ZWATER__Ph10_Ax_3D_DISCO_C_original_glcm_ClusterProminence	.871											
ZWATER__Ph10_Ax_3D_DISCO_C_original_glcm_Contrast	.870											
ZWATER__Ph10_Ax_3D_DISCO_C_original_firstorder_90Percentile	.831	.440										
ZWATER__Ph10_Ax_3D_DISCO_C_original_glcm_JointAverage	.828											
ZWATER__Ph10_Ax_3D_DISCO_C_original_glcm_SumAverage	.828											
ZWATER__Ph10_Ax_3D_DISCO_C_original_glcm_DifferenceAverage	.796	.530										
ZWATER__Ph10_Ax_3D_DISCO_C_original_firstorder_Range	.787											
ZWATER__Ph10_Ax_3D_DISCO_C_original_firstorder_Maximum	.784	.413										
ZWATER__Ph10_Ax_3D_DISCO_C_original_firstorder_RootMeanSquared	.776	.418										
ZWATER__Ph10_Ax_3D_DISCO_C_original_firstorder_Mean	.747	.414				.436						
ZWATER__Ph10_Ax_3D_DISCO_C_original_firstorder_Median	.725					.437						
ZWATER__Ph10_Ax_3D_DISCO_C_original_glrlm_RunEntropy	.719	.561										
ZWATER__Ph10_Ax_3D_DISCO_C_original_firstorder_Entropy	.689	.650										
ZWATER__Ph10_Ax_3D_DISCO_C_original_glcm_SumEntropy	.680	.620										
ZWATER__Ph10_Ax_3D_DISCO_C_original_glszm_SizeZoneNonUniformityN	.654	.643										
ZWATER__Ph10_Ax_3D_DISCO_C_original_glcm_JointEntropy	.622	.556		.416								
ZWATER__Ph10_Ax_3D_DISCO_C_original_ngtdm_Contrast	.584	.415		-.482								
ZWATER__Ph10_Ax_3D_DISCO_C_original_gldm_LargeDependenceHighGray	.569			.409				-.519				
ZWATER__Ph10_Ax_3D_DISCO_C_original_gldm_LargeDependenceEmphasis		-.921										
ZWATER__Ph10_Ax_3D_DISCO_C_original_glrlm_LongRunEmphasis		-.916										
ZWATER__Ph10_Ax_3D_DISCO_C_original_glrlm_RunVariance		-.915										
ZWATER__Ph10_Ax_3D_DISCO_C_original_gldm_DependenceVariance		-.897										
ZWATER__Ph10_Ax_3D_DISCO_C_original_glrlm_ShortRunEmphasis		.895										
ZWATER__Ph10_Ax_3D_DISCO_C_original_glrlm_RunPercentage		.893										
ZSco03	.415	.881										
ZWATER__Ph10_Ax_3D_DISCO_C_original_glcm_MaximumProbability		-.861										
ZWATER__Ph10_Ax_3D_DISCO_C_original_glcm_Idm	-.478	-.859										
ZWATER__Ph10_Ax_3D_DISCO_C_original_glcm_JointEnergy		-.857										
ZWATER__Ph10_Ax_3D_DISCO_C_original_firstorder_Uniformity		-.850										
ZSco02	-.401	-.838										
ZWATER__Ph10_Ax_3D_DISCO_C_original_glcm_InverseVariance	-.510	-.831										
ZWATER__Ph10_Ax_3D_DISCO_C_original_glcm_Id	-.540	-.817										
ZSco04	-.459	-.772										
ZWATER__Ph10_Ax_3D_DISCO_C_original_glszm_ZonePercentage	.557	.766										
ZWATER__Ph10_Ax_3D_DISCO_C_original_gldm_SmallDependenceEmphasis	.594	.735										
ZWATER__Ph10_Ax_3D_DISCO_C_original_gldm_LargeDependenceLowGrayL		-.694									.404	
ZWATER__Ph10_Ax_3D_DISCO_C_original_glszm_SmallAreaEmphasis	.611	.692										
ZWATER__Ph10_Ax_3D_DISCO_C_original_glcm_DifferenceEntropy	.685	.688										
ZSco01	.622	.672										
ZWATER__Ph10_Ax_3D_DISCO_C_original_glcm_Imc2		.631										
ZWATER__Ph10_Ax_3D_DISCO_C_original_glszm_LowGrayLevelZoneEmphas		-.625		-.612								
ZWATER__Ph10_Ax_3D_DISCO_C_original_firstorder_Kurtosis		-.494										
ZWATER__Ph10_Ax_3D_DISCO_C_original_shape_SurfaceArea			.980									
ZWATER__Ph10_Ax_3D_DISCO_C_original_glrlm_RunLengthNonUniformity			.976									
ZWATER__Ph10_Ax_3D_DISCO_C_original_glszm_GrayLevelNonUniformity			.975									
ZWATER__Ph10_Ax_3D_DISCO_C_original_gldm_DependenceNonUniformity			.972									
ZWATER__Ph10_Ax_3D_DISCO_C_original_shape_VoxelVolume			.955									
ZWATER__Ph10_Ax_3D_DISCO_C_original_shape_MeshVolume			.955									
ZWATER__Ph10_Ax_3D_DISCO_C_original_glszm_SizeZoneNonUniformity			.850									
ZWATER__Ph10_Ax_3D_DISCO_C_original_shape_Maximum2DDiameterColum			.843									
ZWATER__Ph10_Ax_3D_DISCO_C_original_shape_LeastAxisLength			.839									
ZWATER__Ph10_Ax_3D_DISCO_C_original_shape_MajorAxisLength			.832									
ZWATER__Ph10_Ax_3D_DISCO_C_original_glrlm_GrayLevelNonUniformity			.819									
ZWATER__Ph10_Ax_3D_DISCO_C_original_shape_MinorAxisLength			.819									
ZWATER__Ph10_Ax_3D_DISCO_C_original_shape_Maximum3DDiameter			.814									
ZWATER__Ph10_Ax_3D_DISCO_C_original_shape_Maximum2DDiameterRow			.812									
ZWATER__Ph10_Ax_3D_DISCO_C_original_shape_Maximum2DDiameterSlice			.806									
ZWATER__Ph10_Ax_3D_DISCO_C_original_gldm_GrayLevelNonUniformity			.805									
ZWATER__Ph10_Ax_3D_DISCO_C_original_ngtdm_Busyness			.798									
ZWATER__Ph10_Ax_3D_DISCO_C_original_firstorder_TotalEnergy			.793							.415		
ZWATER__Ph10_Ax_3D_DISCO_C_original_firstorder_Energy			.790							.416		
ZWATER__Ph10_Ax_3D_DISCO_C_original_gldm_SmallDependenceLowGrayL				-.871								
ZWATER__Ph10_Ax_3D_DISCO_C_original_ngtdm_Coarseness				-.834								
ZWATER__Ph10_Ax_3D_DISCO_C_original_glrlm_ShortRunLowGrayLevelEm				-.834								
ZWATER__Ph10_Ax_3D_DISCO_C_original_gldm_LowGrayLevelEmphasis				-.827								
ZWATER__Ph10_Ax_3D_DISCO_C_original_glrlm_LowGrayLevelRunEmphasi				-.822								
ZWATER__Ph10_Ax_3D_DISCO_C_original_glrlm_LongRunLowGrayLevelEmp		-.502		-.749								
ZWATER__Ph10_Ax_3D_DISCO_C_original_glcm_Idmn				.721								
ZWATER__Ph10_Ax_3D_DISCO_C_original_shape_SurfaceVolumeRatio			-.409	-.693								
ZWATER__Ph10_Ax_3D_DISCO_C_original_glcm_Idn			.409	.680								
ZWATER__Ph10_Ax_3D_DISCO_C_original_glcm_Imc1				.668								
ZWATER__Ph10_Ax_3D_DISCO_C_original_gldm_DependenceEntropy	.544			.613								
ZWATER__Ph10_Ax_3D_DISCO_C_original_glszm_ZoneEntropy	.512			.593								
ZWATER__Ph10_Ax_3D_DISCO_C_original_glszm_SmallAreaLowGrayLevelE		-.566		-.585								
ZWATER__Ph10_Ax_3D_DISCO_C_original_ngtdm_Strength	.402			-.504								
ZWATER__Ph10_Ax_3D_DISCO_C_original_glszm_LargeAreaHighGrayLevel					.961							
ZWATER__Ph10_Ax_3D_DISCO_C_original_glszm_ZoneVariance					.950							
ZWATER__Ph10_Ax_3D_DISCO_C_original_glszm_LargeAreaEmphasis					.950							
ZWATER__Ph10_Ax_3D_DISCO_C_original_glszm_LargeAreaLowGrayLevelE					.878							
ZWATER__Ph10_Ax_3D_DISCO_C_original_firstorder_Minimum						.743						
ZWATER__Ph10_Ax_3D_DISCO_C_original_firstorder_10Percentile	.457					.739						
ZWATER__Ph10_Ax_3D_DISCO_C_original_glcm_MCC							.878					
ZWATER__Ph10_Ax_3D_DISCO_C_original_glcm_Correlation				.402			.764					
ZWATER__Ph10_Ax_3D_DISCO_C_original_glcm_ClusterShade								.823				
ZWATER__Ph10_Ax_3D_DISCO_C_original_firstorder_Skewness								.795				
ZWATER__Ph10_Ax_3D_DISCO_C_original_shape_Flatness									.920			
ZWATER__Ph10_Ax_3D_DISCO_C_original_shape_Elongation									.879			
ZWATER__Ph10_Ax_3D_DISCO_C_original_shape_Sphericity			-.431						.603			

Extraction Method: Principal Component Analysis. 
 Rotation Method: Varimax with Kaiser Normalization.a	
a. Rotation converged in 11 iterations.	
Factor loadings < 0.4 were surpressed and are displayed as blank spaces. 
