# Supplementary material for: Combination of ultrafast dynamic contrast-enhanced MRI-based radiomics and artificial neural network in assessing BI-RADS 4 breast lesions: Potential to avoid unnecessary biopsies
Source: Front Oncol. 2023 Feb 1;13:1074060. doi: 10.3389/fonc.2023.1074060 (PMC9929366; doi:10.3389/fonc.2023.1074060)
Supplement: Supplementary file 3 [file Table_3.doc]

Supplementary materials 3

PCA analysis of the radiomics based on DISCO-15


Total Variance Explained	
Component	Initial Eigenvalues	Extraction Sums of Squared Loadings	Rotation Sums of Squared Loadings	
	Total	% of Variance	Cumulative %	Total	% of Variance	Cumulative %	Total	% of Variance	Cumulative %	
1	47.389	44.289	44.289	47.389	44.289	44.289	30.466	28.473	28.473	
2	24.294	22.705	66.994	24.294	22.705	66.994	22.216	20.763	49.236	
3	7.786	7.276	74.270	7.786	7.276	74.270	17.124	16.004	65.240	
4	6.921	6.469	80.739	6.921	6.469	80.739	13.371	12.496	77.736	
5	4.266	3.987	84.726	4.266	3.987	84.726	4.159	3.887	81.623	
6	3.213	3.003	87.729	3.213	3.003	87.729	3.642	3.403	85.027	
7	2.261	2.113	89.843	2.261	2.113	89.843	2.634	2.462	87.488	
8	1.872	1.750	91.593	1.872	1.750	91.593	2.624	2.453	89.941	
9	1.505	1.407	92.999	1.505	1.407	92.999	2.186	2.043	91.984	
10	1.218	1.139	94.138	1.218	1.139	94.138	1.945	1.817	93.802	
11	1.133	1.059	95.197	1.133	1.059	95.197	1.493	1.395	95.197	
12	.871	.814	96.011							
13	.806	.754	96.764							
14	.619	.579	97.343							
15	.424	.396	97.739							
16	.374	.349	98.088							
17	.288	.269	98.357							
18	.230	.215	98.572							
19	.199	.186	98.758							
20	.160	.150	98.908							
21	.155	.145	99.052							
22	.146	.137	99.189							
23	.131	.123	99.312							
24	.114	.106	99.418							
25	.078	.073	99.491							
26	.070	.066	99.557							
27	.059	.055	99.612							
28	.053	.050	99.662							
29	.046	.043	99.705							
30	.037	.034	99.739							
31	.031	.029	99.768							
32	.030	.028	99.796							
33	.026	.024	99.820							
34	.022	.021	99.841							
35	.020	.019	99.860							
36	.017	.016	99.876							
37	.015	.014	99.890							
38	.013	.012	99.902							
39	.012	.011	99.913							
40	.010	.010	99.923							
41	.009	.008	99.931							
42	.008	.008	99.939							
43	.008	.007	99.946							
44	.007	.007	99.953							
45	.006	.006	99.959							
46	.005	.005	99.964							
47	.005	.004	99.968							
48	.005	.004	99.973							
49	.004	.004	99.977							
50	.003	.003	99.980							
51	.003	.003	99.982							
52	.003	.002	99.985							
53	.002	.002	99.987							
54	.002	.002	99.989							
55	.002	.001	99.990							
56	.001	.001	99.992							
57	.001	.001	99.993							
58	.001	.001	99.994							
59	.001	.001	99.995							
60	.001	.001	99.995							
61	.001	.001	99.996							
62	.001	.001	99.997							
63	.001	.000	99.997							
64	.000	.000	99.998							
65	.000	.000	99.998							
66	.000	.000	99.998							
67	.000	.000	99.998							
68	.000	.000	99.999							
69	.000	.000	99.999							
70	.000	.000	99.999							
71	.000	.000	99.999							
72	.000	.000	99.999							
73	.000	9.900E-5	100.000							
74	9.780E-5	9.140E-5	100.000							
75	7.250E-5	6.776E-5	100.000							
76	6.435E-5	6.014E-5	100.000							
77	5.223E-5	4.881E-5	100.000							
78	4.439E-5	4.149E-5	100.000							
79	3.600E-5	3.364E-5	100.000							
80	2.701E-5	2.524E-5	100.000							
81	2.401E-5	2.244E-5	100.000							
82	1.559E-5	1.457E-5	100.000							
83	1.192E-5	1.114E-5	100.000							
84	1.063E-5	9.938E-6	100.000							
85	9.191E-6	8.589E-6	100.000							
86	7.165E-6	6.696E-6	100.000							
87	6.369E-6	5.952E-6	100.000							
88	5.358E-6	5.008E-6	100.000							
89	3.815E-6	3.565E-6	100.000							
90	3.687E-6	3.445E-6	100.000							
91	3.060E-6	2.860E-6	100.000							
92	2.132E-6	1.992E-6	100.000							
93	1.850E-6	1.729E-6	100.000							
94	1.284E-6	1.200E-6	100.000							
95	3.432E-7	3.207E-7	100.000							
96	2.171E-7	2.029E-7	100.000							
97	1.692E-7	1.581E-7	100.000							
98	1.263E-7	1.181E-7	100.000							
99	8.174E-8	7.640E-8	100.000							
100	4.663E-8	4.358E-8	100.000							
101	3.519E-8	3.289E-8	100.000							
102	1.366E-8	1.276E-8	100.000							
103	6.790E-9	6.346E-9	100.000							
104	4.864E-10	4.546E-10	100.000							
105	9.225E-16	8.622E-16	100.000							
106	-4.744E-16	-4.434E-16	100.000							
107	-7.594E-16	-7.097E-16	100.000							

Extraction Method: Principal Component Analysis.	


Rotated Component Matrixa	
	Component	
	1	2	3	4	5	6	7	8	9	10	11	
ZWATER__Ph15_Ax_3D_DISCO_C_original_glszm_GrayLevelVariance	.941											
ZWATER__Ph15_Ax_3D_DISCO_C_original_glrlm_GrayLevelVariance	.938											
ZWATER__Ph15_Ax_3D_DISCO_C_original_firstorder_Variance	.937											
ZWATER__Ph15_Ax_3D_DISCO_C_original_gldm_GrayLevelVariance	.937											
ZWATER__Ph15_Ax_3D_DISCO_C_original_gldm_SmallDependenceHighGray	.926											
ZWATER__Ph15_Ax_3D_DISCO_C_original_glcm_SumSquares	.925											
ZWATER__Ph15_Ax_3D_DISCO_C_original_glcm_ClusterTendency	.923											
ZWATER__Ph15_Ax_3D_DISCO_C_original_glszm_SmallAreaHighGrayLevel	.911											
ZWATER__Ph15_Ax_3D_DISCO_C_original_ngtdm_Complexity	.898											
ZWATER__Ph15_Ax_3D_DISCO_C_original_glcm_Autocorrelation	.898											
ZWATER__Ph15_Ax_3D_DISCO_C_original_glrlm_ShortRunHighGrayLevelE	.895											
ZWATER__Ph15_Ax_3D_DISCO_C_original_glszm_HighGrayLevelZoneEmpha	.892											
ZWATER__Ph15_Ax_3D_DISCO_C_original_glrlm_HighGrayLevelRunEmphas	.892											
ZWATER__Ph15_Ax_3D_DISCO_C_original_gldm_HighGrayLevelEmphasis	.892											
ZWATER__Ph15_Ax_3D_DISCO_C_original_glrlm_LongRunHighGrayLevelEm	.880											
ZWATER__Ph15_Ax_3D_DISCO_C_original_glcm_DifferenceVariance	.878											
ZWATER__Ph15_Ax_3D_DISCO_C_original_glcm_ClusterProminence	.876											
ZWATER__Ph15_Ax_3D_DISCO_C_original_firstorder_MeanAbsoluteDevia	.856	.453										
ZWATER__Ph15_Ax_3D_DISCO_C_original_firstorder_RobustMeanAbsolut	.847	.445										
ZWATER__Ph15_Ax_3D_DISCO_C_original_firstorder_InterquartileRang	.842	.444										
ZWATER__Ph15_Ax_3D_DISCO_C_original_glcm_Contrast	.827	.457										
ZWATER__Ph15_Ax_3D_DISCO_C_original_glcm_SumAverage	.811											
ZWATER__Ph15_Ax_3D_DISCO_C_original_glcm_JointAverage	.811											
ZWATER__Ph15_Ax_3D_DISCO_C_original_firstorder_90Percentile	.776	.465										
ZWATER__Ph15_Ax_3D_DISCO_C_original_firstorder_Range	.768											
ZWATER__Ph15_Ax_3D_DISCO_C_original_firstorder_Maximum	.749	.444										
ZWATER__Ph15_Ax_3D_DISCO_C_original_glcm_DifferenceAverage	.725	.629										
ZWATER__Ph15_Ax_3D_DISCO_C_original_firstorder_RootMeanSquared	.715	.417				.476						
ZWATER__Ph15_Ax_3D_DISCO_C_original_glrlm_RunEntropy	.707	.542										
ZWATER__Ph15_Ax_3D_DISCO_C_original_firstorder_Mean	.686	.406				.518						
ZWATER__Ph15_Ax_3D_DISCO_C_original_firstorder_Entropy	.679	.637										
ZWATER__Ph15_Ax_3D_DISCO_C_original_firstorder_Median	.675					.521						
ZWATER__Ph15_Ax_3D_DISCO_C_original_glcm_SumEntropy	.673	.590										
ZWATER__Ph15_Ax_3D_DISCO_C_original_glcm_JointEntropy	.604	.473		.520								
ZWATER__Ph15_Ax_3D_DISCO_C_original_gldm_LargeDependenceHighGray	.604			.404			-.433					
ZWATER__Ph15_Ax_3D_DISCO_C_original_glrlm_RunPercentage		.898										
ZWATER__Ph15_Ax_3D_DISCO_C_original_glrlm_ShortRunEmphasis		.897										
ZSco07		.895										
ZWATER__Ph15_Ax_3D_DISCO_C_original_glrlm_LongRunEmphasis		-.893										
ZWATER__Ph15_Ax_3D_DISCO_C_original_gldm_LargeDependenceEmphasis		-.893										
ZWATER__Ph15_Ax_3D_DISCO_C_original_glrlm_RunVariance		-.888										
ZWATER__Ph15_Ax_3D_DISCO_C_original_glcm_Idm	-.424	-.888										
ZWATER__Ph15_Ax_3D_DISCO_C_original_gldm_DependenceVariance		-.884										
ZWATER__Ph15_Ax_3D_DISCO_C_original_glcm_InverseVariance	-.439	-.880										
ZWATER__Ph15_Ax_3D_DISCO_C_original_glcm_Id	-.482	-.858										
ZWATER__Ph15_Ax_3D_DISCO_C_original_glszm_ZonePercentage	.450	.852										
ZWATER__Ph15_Ax_3D_DISCO_C_original_gldm_SmallDependenceEmphasis	.489	.823										
ZWATER__Ph15_Ax_3D_DISCO_C_original_firstorder_Uniformity	-.431	-.788										
ZSco06	-.440	-.778										
ZSco05	.506	.767										
ZWATER__Ph15_Ax_3D_DISCO_C_original_glszm_SmallAreaEmphasis	.538	.763										
ZWATER__Ph15_Ax_3D_DISCO_C_original_glcm_MaximumProbability		-.736		-.532								
ZWATER__Ph15_Ax_3D_DISCO_C_original_glszm_SizeZoneNonUniformityN	.560	.734										
ZWATER__Ph15_Ax_3D_DISCO_C_original_glcm_DifferenceEntropy	.639	.733										
ZWATER__Ph15_Ax_3D_DISCO_C_original_glcm_JointEnergy		-.723		-.525								
ZSco08	-.494	-.701										
ZWATER__Ph15_Ax_3D_DISCO_C_original_glcm_Imc2		.582										
ZWATER__Ph15_Ax_3D_DISCO_C_original_gldm_LargeDependenceLowGrayL		-.555									.538	
ZWATER__Ph15_Ax_3D_DISCO_C_original_ngtdm_Contrast	.500	.545										
ZWATER__Ph15_Ax_3D_DISCO_C_original_ngtdm_Strength		.416										
ZWATER__Ph15_Ax_3D_DISCO_C_original_shape_SurfaceArea			.977									
ZWATER__Ph15_Ax_3D_DISCO_C_original_gldm_DependenceNonUniformity			.975									
ZWATER__Ph15_Ax_3D_DISCO_C_original_glrlm_RunLengthNonUniformity			.967									
ZWATER__Ph15_Ax_3D_DISCO_C_original_glszm_GrayLevelNonUniformity			.965									
ZWATER__Ph15_Ax_3D_DISCO_C_original_shape_VoxelVolume			.943									
ZWATER__Ph15_Ax_3D_DISCO_C_original_shape_MeshVolume			.943									
ZWATER__Ph15_Ax_3D_DISCO_C_original_glszm_SizeZoneNonUniformity			.865									
ZWATER__Ph15_Ax_3D_DISCO_C_original_shape_Maximum2DDiameterColum			.845									
ZWATER__Ph15_Ax_3D_DISCO_C_original_shape_MajorAxisLength			.835									
ZWATER__Ph15_Ax_3D_DISCO_C_original_shape_LeastAxisLength			.835									
ZWATER__Ph15_Ax_3D_DISCO_C_original_shape_MinorAxisLength			.820									
ZWATER__Ph15_Ax_3D_DISCO_C_original_shape_Maximum3DDiameter			.818									
ZWATER__Ph15_Ax_3D_DISCO_C_original_shape_Maximum2DDiameterRow			.813									
ZWATER__Ph15_Ax_3D_DISCO_C_original_shape_Maximum2DDiameterSlice			.811									
ZWATER__Ph15_Ax_3D_DISCO_C_original_glrlm_GrayLevelNonUniformity			.805									
ZWATER__Ph15_Ax_3D_DISCO_C_original_ngtdm_Busyness			.799									
ZWATER__Ph15_Ax_3D_DISCO_C_original_firstorder_TotalEnergy			.791									
ZWATER__Ph15_Ax_3D_DISCO_C_original_gldm_GrayLevelNonUniformity			.791									
ZWATER__Ph15_Ax_3D_DISCO_C_original_firstorder_Energy			.788									
ZWATER__Ph15_Ax_3D_DISCO_C_original_firstorder_Kurtosis		-.414	.456									
ZWATER__Ph15_Ax_3D_DISCO_C_original_glrlm_ShortRunLowGrayLevelEm				-.892								
ZWATER__Ph15_Ax_3D_DISCO_C_original_glrlm_LowGrayLevelRunEmphasi				-.887								
ZWATER__Ph15_Ax_3D_DISCO_C_original_gldm_LowGrayLevelEmphasis				-.885								
ZWATER__Ph15_Ax_3D_DISCO_C_original_gldm_SmallDependenceLowGrayL				-.866								
ZWATER__Ph15_Ax_3D_DISCO_C_original_glrlm_LongRunLowGrayLevelEmp				-.841								
ZWATER__Ph15_Ax_3D_DISCO_C_original_glszm_LowGrayLevelZoneEmphas				-.831								
ZWATER__Ph15_Ax_3D_DISCO_C_original_glszm_SmallAreaLowGrayLevelE				-.807								
ZWATER__Ph15_Ax_3D_DISCO_C_original_ngtdm_Coarseness				-.795								
ZWATER__Ph15_Ax_3D_DISCO_C_original_glcm_Idmn		-.415		.728								
ZWATER__Ph15_Ax_3D_DISCO_C_original_glcm_Idn		-.460		.684								
ZWATER__Ph15_Ax_3D_DISCO_C_original_gldm_DependenceEntropy	.550			.676								
ZWATER__Ph15_Ax_3D_DISCO_C_original_glszm_ZoneEntropy	.548			.663								
ZWATER__Ph15_Ax_3D_DISCO_C_original_shape_SurfaceVolumeRatio			-.403	-.659								
ZWATER__Ph15_Ax_3D_DISCO_C_original_glcm_Imc1				.596								
ZWATER__Ph15_Ax_3D_DISCO_C_original_glszm_LargeAreaHighGrayLevel					.949							
ZWATER__Ph15_Ax_3D_DISCO_C_original_glszm_ZoneVariance					.929							
ZWATER__Ph15_Ax_3D_DISCO_C_original_glszm_LargeAreaEmphasis					.929							
ZWATER__Ph15_Ax_3D_DISCO_C_original_glszm_LargeAreaLowGrayLevelE			.444		.743							
ZWATER__Ph15_Ax_3D_DISCO_C_original_firstorder_10Percentile						.788						
ZWATER__Ph15_Ax_3D_DISCO_C_original_firstorder_Minimum						.752						
ZWATER__Ph15_Ax_3D_DISCO_C_original_firstorder_Skewness							.831					
ZWATER__Ph15_Ax_3D_DISCO_C_original_glcm_ClusterShade	-.489						.665					
ZWATER__Ph15_Ax_3D_DISCO_C_original_shape_Flatness								.916				
ZWATER__Ph15_Ax_3D_DISCO_C_original_shape_Elongation								.843				
ZWATER__Ph15_Ax_3D_DISCO_C_original_shape_Sphericity			-.441					.599				
ZWATER__Ph15_Ax_3D_DISCO_C_original_glcm_MCC										.847		
ZWATER__Ph15_Ax_3D_DISCO_C_original_glcm_Correlation				.532						.567		

Extraction Method: Principal Component Analysis. 
 Rotation Method: Varimax with Kaiser Normalization.a	
a. Rotation converged in 11 iterations.	
Factor loadings < 0.4 were surpressed and are displayed as blank spaces. 
