# Supplementary material for: Combination of ultrafast dynamic contrast-enhanced MRI-based radiomics and artificial neural network in assessing BI-RADS 4 breast lesions: Potential to avoid unnecessary biopsies
Source: Front Oncol. 2023 Feb 1;13:1074060. doi: 10.3389/fonc.2023.1074060 (PMC9929366; doi:10.3389/fonc.2023.1074060)
Supplement: Supplementary file 4 [file Table_4.doc]

Supplementary materials 4

PCA analysis of the radiomics based on DISCO-Combined


Total Variance Explained	
Component	Initial Eigenvalues	Extraction Sums of Squared Loadings	Rotation Sums of Squared Loadings	
	Total	% of Variance	Cumulative %	Total	% of Variance	Cumulative %	Total	% of Variance	Cumulative %	
1	91.431	42.725	42.725	91.431	42.725	42.725	59.772	27.931	27.931	
2	45.475	21.250	63.975	45.475	21.250	63.975	41.049	19.182	47.112	
3	14.767	6.901	70.876	14.767	6.901	70.876	33.656	15.727	62.839	
4	13.974	6.530	77.406	13.974	6.530	77.406	23.881	11.159	73.999	
5	8.253	3.856	81.262	8.253	3.856	81.262	8.451	3.949	77.948	
6	6.285	2.937	84.199	6.285	2.937	84.199	5.921	2.767	80.715	
7	5.129	2.397	86.595	5.129	2.397	86.595	5.165	2.414	83.129	
8	4.582	2.141	88.736	4.582	2.141	88.736	5.075	2.371	85.500	
9	3.675	1.717	90.454	3.675	1.717	90.454	4.489	2.098	87.598	
10	3.022	1.412	91.866	3.022	1.412	91.866	3.627	1.695	89.292	
11	2.314	1.081	92.947	2.314	1.081	92.947	3.174	1.483	90.776	
12	1.994	.932	93.879	1.994	.932	93.879	3.096	1.447	92.222	
13	1.678	.784	94.663	1.678	.784	94.663	2.843	1.328	93.551	
14	1.608	.751	95.414	1.608	.751	95.414	2.264	1.058	94.609	
15	1.377	.643	96.057	1.377	.643	96.057	2.125	.993	95.602	
16	1.125	.526	96.583	1.125	.526	96.583	2.100	.981	96.583	
17	.885	.413	96.996							
18	.823	.385	97.381							
19	.680	.318	97.699							
20	.499	.233	97.932							
21	.477	.223	98.155							
22	.381	.178	98.333							
23	.347	.162	98.495							
24	.311	.145	98.641							
25	.269	.126	98.766							
26	.259	.121	98.888							
27	.234	.109	98.997							
28	.206	.096	99.093							
29	.181	.085	99.178							
30	.155	.073	99.250							
31	.151	.070	99.321							
32	.118	.055	99.376							
33	.110	.051	99.427							
34	.099	.046	99.473							
35	.095	.044	99.518							
36	.082	.038	99.556							
37	.073	.034	99.590							
38	.072	.033	99.623							
39	.066	.031	99.654							
40	.058	.027	99.681							
41	.052	.024	99.706							
42	.050	.024	99.729							
43	.047	.022	99.751							
44	.040	.019	99.770							
45	.038	.018	99.788							
46	.035	.016	99.804							
47	.033	.015	99.819							
48	.030	.014	99.833							
49	.028	.013	99.846							
50	.026	.012	99.858							
51	.024	.011	99.870							
52	.022	.010	99.880							
53	.020	.009	99.889							
54	.019	.009	99.898							
55	.018	.008	99.907							
56	.015	.007	99.914							
57	.014	.007	99.920							
58	.013	.006	99.926							
59	.012	.006	99.932							
60	.011	.005	99.937							
61	.011	.005	99.942							
62	.010	.005	99.947							
63	.009	.004	99.951							
64	.008	.004	99.955							
65	.008	.004	99.959							
66	.007	.003	99.962							
67	.007	.003	99.965							
68	.006	.003	99.968							
69	.006	.003	99.971							
70	.005	.002	99.973							
71	.005	.002	99.975							
72	.004	.002	99.977							
73	.004	.002	99.979							
74	.004	.002	99.981							
75	.003	.002	99.982							
76	.003	.001	99.984							
77	.003	.001	99.985							
78	.002	.001	99.986							
79	.002	.001	99.987							
80	.002	.001	99.988							
81	.002	.001	99.989							
82	.002	.001	99.990							
83	.002	.001	99.991							
84	.002	.001	99.992							
85	.001	.001	99.992							
86	.001	.001	99.993							
87	.001	.001	99.993							
88	.001	.000	99.994							
89	.001	.000	99.994							
90	.001	.000	99.995							
91	.001	.000	99.995							
92	.001	.000	99.995							
93	.001	.000	99.996							
94	.001	.000	99.996							
95	.001	.000	99.997							
96	.001	.000	99.997							
97	.001	.000	99.997							
98	.001	.000	99.997							
99	.001	.000	99.998							
100	.000	.000	99.998							
101	.000	.000	99.998							
102	.000	.000	99.998							
103	.000	.000	99.998							
104	.000	.000	99.998							
105	.000	.000	99.999							
106	.000	.000	99.999							
107	.000	.000	99.999							
108	.000	9.959E-5	99.999							
109	.000	9.363E-5	99.999							
110	.000	8.483E-5	99.999							
111	.000	7.446E-5	99.999							
112	.000	7.308E-5	99.999							
113	.000	6.518E-5	99.999							
114	.000	6.211E-5	99.999							
115	.000	5.702E-5	99.999							
116	.000	5.366E-5	99.999							
117	.000	5.105E-5	100.000							
118	9.654E-5	4.511E-5	100.000							
119	8.816E-5	4.120E-5	100.000							
120	8.500E-5	3.972E-5	100.000							
121	7.006E-5	3.274E-5	100.000							
122	6.620E-5	3.093E-5	100.000							
123	5.817E-5	2.718E-5	100.000							
124	5.337E-5	2.494E-5	100.000							
125	4.611E-5	2.155E-5	100.000							
126	4.371E-5	2.043E-5	100.000							
127	3.651E-5	1.706E-5	100.000							
128	3.421E-5	1.599E-5	100.000							
129	3.064E-5	1.432E-5	100.000							
130	2.851E-5	1.332E-5	100.000							
131	2.764E-5	1.292E-5	100.000							
132	2.500E-5	1.168E-5	100.000							
133	2.375E-5	1.110E-5	100.000							
134	2.108E-5	9.850E-6	100.000							
135	1.827E-5	8.537E-6	100.000							
136	1.427E-5	6.667E-6	100.000							
137	1.373E-5	6.416E-6	100.000							
138	1.230E-5	5.749E-6	100.000							
139	1.101E-5	5.144E-6	100.000							
140	1.043E-5	4.875E-6	100.000							
141	9.855E-6	4.605E-6	100.000							
142	9.029E-6	4.219E-6	100.000							
143	7.268E-6	3.396E-6	100.000							
144	6.183E-6	2.889E-6	100.000							
145	4.878E-6	2.279E-6	100.000							
146	4.361E-6	2.038E-6	100.000							
147	3.692E-6	1.725E-6	100.000							
148	3.577E-6	1.671E-6	100.000							
149	3.330E-6	1.556E-6	100.000							
150	2.947E-6	1.377E-6	100.000							
151	2.590E-6	1.210E-6	100.000							
152	2.384E-6	1.114E-6	100.000							
153	2.231E-6	1.042E-6	100.000							
154	1.725E-6	8.061E-7	100.000							
155	1.631E-6	7.621E-7	100.000							
156	1.518E-6	7.096E-7	100.000							
157	1.238E-6	5.785E-7	100.000							
158	1.143E-6	5.339E-7	100.000							
159	8.746E-7	4.087E-7	100.000							
160	7.666E-7	3.582E-7	100.000							
161	4.828E-7	2.256E-7	100.000							
162	4.053E-7	1.894E-7	100.000							
163	3.374E-7	1.576E-7	100.000							
164	2.424E-7	1.133E-7	100.000							
165	1.962E-7	9.167E-8	100.000							
166	1.644E-7	7.684E-8	100.000							
167	1.267E-7	5.921E-8	100.000							
168	1.105E-7	5.161E-8	100.000							
169	9.760E-8	4.561E-8	100.000							
170	7.454E-8	3.483E-8	100.000							
171	4.395E-8	2.054E-8	100.000							
172	4.081E-8	1.907E-8	100.000							
173	3.174E-8	1.483E-8	100.000							
174	2.970E-8	1.388E-8	100.000							
175	2.121E-8	9.912E-9	100.000							
176	1.377E-8	6.436E-9	100.000							
177	1.160E-8	5.419E-9	100.000							
178	6.517E-9	3.046E-9	100.000							
179	3.045E-9	1.423E-9	100.000							
180	2.590E-9	1.210E-9	100.000							
181	1.597E-9	7.464E-10	100.000							
182	5.797E-15	2.709E-15	100.000							
183	3.973E-15	1.856E-15	100.000							
184	2.622E-15	1.225E-15	100.000							
185	2.319E-15	1.083E-15	100.000							
186	1.756E-15	8.207E-16	100.000							
187	1.465E-15	6.844E-16	100.000							
188	1.153E-15	5.389E-16	100.000							
189	1.091E-15	5.099E-16	100.000							
190	9.756E-16	4.559E-16	100.000							
191	7.272E-16	3.398E-16	100.000							
192	5.915E-16	2.764E-16	100.000							
193	4.997E-16	2.335E-16	100.000							
194	4.780E-16	2.234E-16	100.000							
195	3.308E-16	1.546E-16	100.000							
196	2.858E-16	1.336E-16	100.000							
197	2.294E-16	1.072E-16	100.000							
198	1.277E-16	5.967E-17	100.000							
199	1.935E-17	9.044E-18	100.000							
200	-4.119E-17	-1.925E-17	100.000							
201	-1.257E-16	-5.873E-17	100.000							
202	-2.047E-16	-9.566E-17	100.000							
203	-2.806E-16	-1.311E-16	100.000							
204	-4.674E-16	-2.184E-16	100.000							
205	-5.451E-16	-2.547E-16	100.000							
206	-6.866E-16	-3.208E-16	100.000							
207	-8.097E-16	-3.784E-16	100.000							
208	-1.064E-15	-4.971E-16	100.000							
209	-1.259E-15	-5.885E-16	100.000							
210	-1.491E-15	-6.965E-16	100.000							
211	-1.529E-15	-7.146E-16	100.000							
212	-2.403E-15	-1.123E-15	100.000							
213	-7.005E-15	-3.273E-15	100.000							
214	-1.823E-14	-8.518E-15	100.000							

Extraction Method: Principal Component Analysis.	


Rotated Component Matrixa	
	Component	
	1	2	3	4	5	6	7	8	9	10	11	12	13	14	15	16	
ZWATER__Ph10_Ax_3D_DISCO_C_original_glszm_GrayLevelVariance	.948																
ZWATER__Ph10_Ax_3D_DISCO_C_original_glrlm_GrayLevelVariance	.945																
ZWATER__Ph10_Ax_3D_DISCO_C_original_firstorder_Variance	.944																
ZWATER__Ph10_Ax_3D_DISCO_C_original_gldm_GrayLevelVariance	.944																
ZWATER__Ph10_Ax_3D_DISCO_C_original_glcm_SumSquares	.938																
ZWATER__Ph10_Ax_3D_DISCO_C_original_glcm_ClusterTendency	.937																
ZWATER__Ph10_Ax_3D_DISCO_C_original_gldm_SmallDependenceHighGray	.924																
ZWATER__Ph15_Ax_3D_DISCO_C_original_glszm_GrayLevelVariance	.914																
ZWATER__Ph15_Ax_3D_DISCO_C_original_glrlm_GrayLevelVariance	.912																
ZWATER__Ph15_Ax_3D_DISCO_C_original_firstorder_Variance	.911																
ZWATER__Ph15_Ax_3D_DISCO_C_original_gldm_GrayLevelVariance	.911																
ZWATER__Ph10_Ax_3D_DISCO_C_original_glszm_SmallAreaHighGrayLevel	.911																
ZWATER__Ph15_Ax_3D_DISCO_C_original_glcm_SumSquares	.896																
ZWATER__Ph15_Ax_3D_DISCO_C_original_glcm_ClusterTendency	.894																
ZWATER__Ph15_Ax_3D_DISCO_C_original_gldm_SmallDependenceHighGray	.894																
ZWATER__Ph10_Ax_3D_DISCO_C_original_glcm_Autocorrelation	.893																
ZWATER__Ph10_Ax_3D_DISCO_C_original_glszm_HighGrayLevelZoneEmpha	.892																
ZWATER__Ph10_Ax_3D_DISCO_C_original_glrlm_ShortRunHighGrayLevelE	.891																
ZWATER__Ph10_Ax_3D_DISCO_C_original_glcm_ClusterProminence	.890																
ZWATER__Ph10_Ax_3D_DISCO_C_original_glrlm_HighGrayLevelRunEmphas	.888																
ZWATER__Ph10_Ax_3D_DISCO_C_original_gldm_HighGrayLevelEmphasis	.887																
ZWATER__Ph10_Ax_3D_DISCO_C_original_ngtdm_Complexity	.884																
ZWATER__Ph15_Ax_3D_DISCO_C_original_glszm_SmallAreaHighGrayLevel	.882																
ZWATER__Ph15_Ax_3D_DISCO_C_original_glcm_Autocorrelation	.878																
ZWATER__Ph15_Ax_3D_DISCO_C_original_glrlm_ShortRunHighGrayLevelE	.874																
ZWATER__Ph10_Ax_3D_DISCO_C_original_glrlm_LongRunHighGrayLevelEm	.874																
ZWATER__Ph15_Ax_3D_DISCO_C_original_gldm_HighGrayLevelEmphasis	.873																
ZWATER__Ph15_Ax_3D_DISCO_C_original_glrlm_HighGrayLevelRunEmphas	.873																
ZWATER__Ph15_Ax_3D_DISCO_C_original_glszm_HighGrayLevelZoneEmpha	.869																
ZWATER__Ph10_Ax_3D_DISCO_C_original_firstorder_MeanAbsoluteDevia	.868																
ZWATER__Ph15_Ax_3D_DISCO_C_original_ngtdm_Complexity	.865																
ZWATER__Ph15_Ax_3D_DISCO_C_original_glrlm_LongRunHighGrayLevelEm	.863																
ZWATER__Ph10_Ax_3D_DISCO_C_original_firstorder_RobustMeanAbsolut	.861																
ZWATER__Ph15_Ax_3D_DISCO_C_original_glcm_ClusterProminence	.860																
ZWATER__Ph10_Ax_3D_DISCO_C_original_glcm_DifferenceVariance	.858																
ZWATER__Ph10_Ax_3D_DISCO_C_original_firstorder_InterquartileRang	.857																
ZWATER__Ph15_Ax_3D_DISCO_C_original_glcm_DifferenceVariance	.854																
ZWATER__Ph15_Ax_3D_DISCO_C_original_firstorder_MeanAbsoluteDevia	.831	.443															
ZWATER__Ph15_Ax_3D_DISCO_C_original_firstorder_RobustMeanAbsolut	.826	.430															
ZWATER__Ph10_Ax_3D_DISCO_C_original_glcm_Contrast	.822																
ZWATER__Ph15_Ax_3D_DISCO_C_original_firstorder_InterquartileRang	.822	.426															
ZWATER__Ph10_Ax_3D_DISCO_C_original_glcm_JointAverage	.806																
ZWATER__Ph10_Ax_3D_DISCO_C_original_glcm_SumAverage	.806																
ZWATER__Ph10_Ax_3D_DISCO_C_original_firstorder_90Percentile	.804	.427															
ZWATER__Ph15_Ax_3D_DISCO_C_original_glcm_Contrast	.796	.435															
ZWATER__Ph15_Ax_3D_DISCO_C_original_glcm_SumAverage	.790																
ZWATER__Ph15_Ax_3D_DISCO_C_original_glcm_JointAverage	.790																
ZWATER__Ph10_Ax_3D_DISCO_C_original_firstorder_Range	.772																
ZWATER__Ph15_Ax_3D_DISCO_C_original_firstorder_90Percentile	.766	.458															
ZWATER__Ph10_Ax_3D_DISCO_C_original_firstorder_Maximum	.765	.417															
ZWATER__Ph10_Ax_3D_DISCO_C_original_glcm_DifferenceAverage	.750	.539															
ZWATER__Ph10_Ax_3D_DISCO_C_original_firstorder_RootMeanSquared	.748	.404				.420											
ZWATER__Ph15_Ax_3D_DISCO_C_original_firstorder_Range	.730	.401															
ZWATER__Ph15_Ax_3D_DISCO_C_original_firstorder_Maximum	.721	.453															
ZWATER__Ph10_Ax_3D_DISCO_C_original_firstorder_Mean	.719					.461											
ZWATER__Ph15_Ax_3D_DISCO_C_original_firstorder_RootMeanSquared	.715	.415				.467											
ZWATER__Ph10_Ax_3D_DISCO_C_original_glrlm_RunEntropy	.701	.522															
ZWATER__Ph10_Ax_3D_DISCO_C_original_firstorder_Median	.696					.462											
ZWATER__Ph15_Ax_3D_DISCO_C_original_glcm_DifferenceAverage	.695	.605															
ZWATER__Ph15_Ax_3D_DISCO_C_original_firstorder_Mean	.688	.405				.507											
ZWATER__Ph15_Ax_3D_DISCO_C_original_firstorder_Median	.681					.503											
ZWATER__Ph15_Ax_3D_DISCO_C_original_glrlm_RunEntropy	.679	.552															
ZWATER__Ph10_Ax_3D_DISCO_C_original_firstorder_Entropy	.669	.617															
ZWATER__Ph10_Ax_3D_DISCO_C_original_glcm_SumEntropy	.665	.584															
ZWATER__Ph15_Ax_3D_DISCO_C_original_firstorder_Entropy	.654	.636															
ZWATER__Ph15_Ax_3D_DISCO_C_original_glcm_SumEntropy	.646	.592															
ZWATER__Ph10_Ax_3D_DISCO_C_original_glcm_JointEntropy	.614	.529		.448													
ZWATER__Ph15_Ax_3D_DISCO_C_original_gldm_LargeDependenceHighGray	.607							-.450									
ZWATER__Ph15_Ax_3D_DISCO_C_original_glcm_JointEntropy	.590	.490		.535													
ZWATER__Ph10_Ax_3D_DISCO_C_original_gldm_LargeDependenceHighGray	.570							-.517									
ZWATER__Ph10_Ax_3D_DISCO_C_original_ngtdm_Contrast	.525	.414		-.430							.438						
ZWATER__Ph10_Ax_3D_DISCO_C_original_gldm_DependenceVariance		-.904															
ZWATER__Ph10_Ax_3D_DISCO_C_original_gldm_LargeDependenceEmphasis		-.903															
ZWATER__Ph10_Ax_3D_DISCO_C_original_glrlm_RunVariance		-.897															
ZWATER__Ph10_Ax_3D_DISCO_C_original_glrlm_LongRunEmphasis		-.896															
ZWATER__Ph10_Ax_3D_DISCO_C_original_glrlm_RunPercentage		.890															
ZWATER__Ph10_Ax_3D_DISCO_C_original_glrlm_ShortRunEmphasis		.886															
ZSco03		.878															
ZWATER__Ph15_Ax_3D_DISCO_C_original_glcm_Idm	-.418	-.857															
ZWATER__Ph15_Ax_3D_DISCO_C_original_glrlm_RunPercentage		.853															
ZWATER__Ph15_Ax_3D_DISCO_C_original_glcm_InverseVariance	-.430	-.852															
ZWATER__Ph15_Ax_3D_DISCO_C_original_glrlm_ShortRunEmphasis		.852															
ZWATER__Ph10_Ax_3D_DISCO_C_original_glcm_Idm	-.452	-.850															
ZSco07		.850															
ZWATER__Ph15_Ax_3D_DISCO_C_original_glrlm_LongRunEmphasis		-.847															
ZWATER__Ph15_Ax_3D_DISCO_C_original_gldm_LargeDependenceEmphasis		-.845															
ZWATER__Ph15_Ax_3D_DISCO_C_original_glrlm_RunVariance		-.840															
ZWATER__Ph10_Ax_3D_DISCO_C_original_glcm_InverseVariance	-.480	-.830															
ZWATER__Ph15_Ax_3D_DISCO_C_original_gldm_DependenceVariance		-.830															
ZWATER__Ph15_Ax_3D_DISCO_C_original_glcm_Id	-.470	-.829															
ZWATER__Ph15_Ax_3D_DISCO_C_original_glszm_ZonePercentage	.436	.813															
ZWATER__Ph10_Ax_3D_DISCO_C_original_glcm_Id	-.510	-.813															
ZWATER__Ph15_Ax_3D_DISCO_C_original_gldm_SmallDependenceEmphasis	.469	.785															
ZWATER__Ph10_Ax_3D_DISCO_C_original_glszm_ZonePercentage	.522	.781															
ZWATER__Ph15_Ax_3D_DISCO_C_original_firstorder_Uniformity	-.429	-.777															
ZSco06	-.437	-.770															
ZWATER__Ph10_Ax_3D_DISCO_C_original_firstorder_Uniformity		-.758															
ZWATER__Ph10_Ax_3D_DISCO_C_original_glcm_MaximumProbability		-.750															
ZSco02		-.749															
ZWATER__Ph10_Ax_3D_DISCO_C_original_gldm_SmallDependenceEmphasis	.557	.746															
ZWATER__Ph10_Ax_3D_DISCO_C_original_glcm_JointEnergy		-.733										.414					
ZWATER__Ph15_Ax_3D_DISCO_C_original_glszm_SmallAreaEmphasis	.515	.732															
ZWATER__Ph15_Ax_3D_DISCO_C_original_glcm_MaximumProbability		-.726		-.497													
ZSco05	.477	.724															
ZWATER__Ph15_Ax_3D_DISCO_C_original_glcm_DifferenceEntropy	.619	.718															
ZWATER__Ph15_Ax_3D_DISCO_C_original_glcm_JointEnergy		-.714		-.499													
ZSco08	-.481	-.714															
ZWATER__Ph15_Ax_3D_DISCO_C_original_glszm_SizeZoneNonUniformityN	.533	.703															
ZSco01	.580	.694															
ZSco04	-.451	-.687															
ZWATER__Ph10_Ax_3D_DISCO_C_original_glcm_DifferenceEntropy	.650	.682															
ZWATER__Ph10_Ax_3D_DISCO_C_original_glszm_SmallAreaEmphasis	.575	.666															
ZWATER__Ph10_Ax_3D_DISCO_C_original_glszm_SizeZoneNonUniformityN	.614	.630															
ZWATER__Ph10_Ax_3D_DISCO_C_original_gldm_LargeDependenceLowGrayL		-.608														.511	
ZWATER__Ph10_Ax_3D_DISCO_C_original_glcm_Imc2		.560							.410								
ZWATER__Ph15_Ax_3D_DISCO_C_original_glcm_Imc2		.532															
ZWATER__Ph15_Ax_3D_DISCO_C_original_ngtdm_Contrast	.498	.500		-.421													
ZWATER__Ph10_Ax_3D_DISCO_C_original_glszm_GrayLevelNonUniformity			.980														
ZWATER__Ph10_Ax_3D_DISCO_C_original_shape_SurfaceArea			.980														
ZWATER__Ph15_Ax_3D_DISCO_C_original_shape_SurfaceArea			.980														
ZWATER__Ph15_Ax_3D_DISCO_C_original_glszm_GrayLevelNonUniformity			.979														
ZWATER__Ph10_Ax_3D_DISCO_C_original_glrlm_RunLengthNonUniformity			.978														
ZWATER__Ph15_Ax_3D_DISCO_C_original_glrlm_RunLengthNonUniformity			.978														
ZWATER__Ph15_Ax_3D_DISCO_C_original_gldm_DependenceNonUniformity			.966														
ZWATER__Ph10_Ax_3D_DISCO_C_original_gldm_DependenceNonUniformity			.965														
ZWATER__Ph10_Ax_3D_DISCO_C_original_shape_VoxelVolume			.960														
ZWATER__Ph15_Ax_3D_DISCO_C_original_shape_VoxelVolume			.960														
ZWATER__Ph10_Ax_3D_DISCO_C_original_shape_MeshVolume			.960														
ZWATER__Ph15_Ax_3D_DISCO_C_original_shape_MeshVolume			.960														
ZWATER__Ph15_Ax_3D_DISCO_C_original_glrlm_GrayLevelNonUniformity			.837														
ZWATER__Ph15_Ax_3D_DISCO_C_original_ngtdm_Busyness			.835														
ZWATER__Ph10_Ax_3D_DISCO_C_original_shape_LeastAxisLength			.835														
ZWATER__Ph15_Ax_3D_DISCO_C_original_shape_LeastAxisLength			.835														
ZWATER__Ph15_Ax_3D_DISCO_C_original_glszm_SizeZoneNonUniformity			.835										.427				
ZWATER__Ph10_Ax_3D_DISCO_C_original_glszm_SizeZoneNonUniformity			.832										.439				
ZWATER__Ph10_Ax_3D_DISCO_C_original_shape_Maximum2DDiameterColum			.831														
ZWATER__Ph15_Ax_3D_DISCO_C_original_shape_Maximum2DDiameterColum			.831														
ZWATER__Ph10_Ax_3D_DISCO_C_original_glrlm_GrayLevelNonUniformity			.831														
ZWATER__Ph10_Ax_3D_DISCO_C_original_shape_MajorAxisLength			.828														
ZWATER__Ph15_Ax_3D_DISCO_C_original_shape_MajorAxisLength			.828														
ZWATER__Ph15_Ax_3D_DISCO_C_original_gldm_GrayLevelNonUniformity			.823														
ZWATER__Ph10_Ax_3D_DISCO_C_original_gldm_GrayLevelNonUniformity			.816														
ZWATER__Ph15_Ax_3D_DISCO_C_original_shape_Maximum3DDiameter			.812														
ZWATER__Ph10_Ax_3D_DISCO_C_original_shape_Maximum3DDiameter			.812														
ZWATER__Ph10_Ax_3D_DISCO_C_original_ngtdm_Busyness			.810														
ZWATER__Ph10_Ax_3D_DISCO_C_original_shape_Maximum2DDiameterRow			.809														
ZWATER__Ph15_Ax_3D_DISCO_C_original_shape_Maximum2DDiameterRow			.809														
ZWATER__Ph10_Ax_3D_DISCO_C_original_shape_MinorAxisLength			.808														
ZWATER__Ph15_Ax_3D_DISCO_C_original_shape_MinorAxisLength			.808														
ZWATER__Ph10_Ax_3D_DISCO_C_original_shape_Maximum2DDiameterSlice			.801														
ZWATER__Ph15_Ax_3D_DISCO_C_original_shape_Maximum2DDiameterSlice			.801														
ZWATER__Ph10_Ax_3D_DISCO_C_original_firstorder_TotalEnergy			.766										.542				
ZWATER__Ph10_Ax_3D_DISCO_C_original_firstorder_Energy			.763										.544				
ZWATER__Ph15_Ax_3D_DISCO_C_original_firstorder_TotalEnergy			.753										.554				
ZWATER__Ph15_Ax_3D_DISCO_C_original_firstorder_Energy			.750										.556				
ZWATER__Ph10_Ax_3D_DISCO_C_original_ngtdm_Coarseness				-.861													
ZWATER__Ph15_Ax_3D_DISCO_C_original_glrlm_ShortRunLowGrayLevelEm				-.860													
ZWATER__Ph15_Ax_3D_DISCO_C_original_gldm_SmallDependenceLowGrayL				-.855													
ZWATER__Ph15_Ax_3D_DISCO_C_original_ngtdm_Coarseness				-.852													
ZWATER__Ph15_Ax_3D_DISCO_C_original_glrlm_LowGrayLevelRunEmphasi				-.851													
ZWATER__Ph15_Ax_3D_DISCO_C_original_gldm_LowGrayLevelEmphasis				-.850													
ZWATER__Ph10_Ax_3D_DISCO_C_original_gldm_SmallDependenceLowGrayL				-.845													
ZWATER__Ph15_Ax_3D_DISCO_C_original_glszm_LowGrayLevelZoneEmphas				-.791													
ZWATER__Ph15_Ax_3D_DISCO_C_original_glrlm_LongRunLowGrayLevelEmp				-.788													
ZWATER__Ph10_Ax_3D_DISCO_C_original_glrlm_ShortRunLowGrayLevelEm				-.774													
ZWATER__Ph15_Ax_3D_DISCO_C_original_glszm_SmallAreaLowGrayLevelE				-.769													
ZWATER__Ph10_Ax_3D_DISCO_C_original_gldm_LowGrayLevelEmphasis				-.767													
ZWATER__Ph10_Ax_3D_DISCO_C_original_glrlm_LowGrayLevelRunEmphasi				-.763													
ZWATER__Ph15_Ax_3D_DISCO_C_original_glcm_Idmn				.743													
ZWATER__Ph15_Ax_3D_DISCO_C_original_gldm_DependenceEntropy	.527			.706													
ZWATER__Ph10_Ax_3D_DISCO_C_original_glcm_Idmn				.705													
ZWATER__Ph15_Ax_3D_DISCO_C_original_shape_SurfaceVolumeRatio				-.701													
ZWATER__Ph10_Ax_3D_DISCO_C_original_shape_SurfaceVolumeRatio				-.701													
ZWATER__Ph15_Ax_3D_DISCO_C_original_glcm_Idn				.695													
ZWATER__Ph10_Ax_3D_DISCO_C_original_glrlm_LongRunLowGrayLevelEmp		-.412		-.688								.403					
ZWATER__Ph15_Ax_3D_DISCO_C_original_glszm_ZoneEntropy	.527			.673													
ZWATER__Ph10_Ax_3D_DISCO_C_original_glcm_Idn		-.412		.662													
ZWATER__Ph10_Ax_3D_DISCO_C_original_glcm_Imc1				.647													
ZWATER__Ph10_Ax_3D_DISCO_C_original_gldm_DependenceEntropy	.550			.646													
ZWATER__Ph15_Ax_3D_DISCO_C_original_glcm_Imc1				.631						-.505							
ZWATER__Ph10_Ax_3D_DISCO_C_original_glszm_ZoneEntropy	.515			.619													
ZWATER__Ph10_Ax_3D_DISCO_C_original_glszm_LowGrayLevelZoneEmphas		-.473		-.562								.548					
ZWATER__Ph10_Ax_3D_DISCO_C_original_glszm_SmallAreaLowGrayLevelE		-.408		-.543								.528					
ZWATER__Ph10_Ax_3D_DISCO_C_original_ngtdm_Strength				-.476													
ZWATER__Ph15_Ax_3D_DISCO_C_original_glszm_LargeAreaHighGrayLevel					.958												
ZWATER__Ph10_Ax_3D_DISCO_C_original_glszm_LargeAreaHighGrayLevel					.955												
ZWATER__Ph10_Ax_3D_DISCO_C_original_glszm_ZoneVariance					.945												
ZWATER__Ph10_Ax_3D_DISCO_C_original_glszm_LargeAreaEmphasis					.945												
ZWATER__Ph15_Ax_3D_DISCO_C_original_glszm_ZoneVariance					.935												
ZWATER__Ph15_Ax_3D_DISCO_C_original_glszm_LargeAreaEmphasis					.935												
ZWATER__Ph10_Ax_3D_DISCO_C_original_glszm_LargeAreaLowGrayLevelE					.873												
ZWATER__Ph15_Ax_3D_DISCO_C_original_glszm_LargeAreaLowGrayLevelE			.469		.755												
ZWATER__Ph15_Ax_3D_DISCO_C_original_firstorder_Minimum						.850											
ZWATER__Ph10_Ax_3D_DISCO_C_original_firstorder_Minimum						.841											
ZWATER__Ph15_Ax_3D_DISCO_C_original_firstorder_10Percentile	.417					.770											
ZWATER__Ph10_Ax_3D_DISCO_C_original_firstorder_10Percentile	.434					.742											
ZWATER__Ph10_Ax_3D_DISCO_C_original_shape_Flatness							.928										
ZWATER__Ph15_Ax_3D_DISCO_C_original_shape_Flatness							.928										
ZWATER__Ph15_Ax_3D_DISCO_C_original_shape_Elongation							.899										
ZWATER__Ph10_Ax_3D_DISCO_C_original_shape_Elongation							.899										
ZWATER__Ph15_Ax_3D_DISCO_C_original_shape_Sphericity			-.425				.578										
ZWATER__Ph10_Ax_3D_DISCO_C_original_shape_Sphericity			-.425				.578										
ZWATER__Ph10_Ax_3D_DISCO_C_original_glcm_ClusterShade								.823									
ZWATER__Ph15_Ax_3D_DISCO_C_original_firstorder_Skewness								.752									
ZWATER__Ph10_Ax_3D_DISCO_C_original_firstorder_Skewness								.741									
ZWATER__Ph15_Ax_3D_DISCO_C_original_glcm_ClusterShade	-.517							.699									
ZWATER__Ph10_Ax_3D_DISCO_C_original_glcm_MCC									.869								
ZWATER__Ph15_Ax_3D_DISCO_C_original_glcm_MCC									.783								
ZWATER__Ph10_Ax_3D_DISCO_C_original_glcm_Correlation				.446					.701								
ZWATER__Ph15_Ax_3D_DISCO_C_original_glcm_Correlation				.549					.593								
ZWATER__Ph15_Ax_3D_DISCO_C_original_ngtdm_Strength										.524							
ZWATER__Ph15_Ax_3D_DISCO_C_original_firstorder_Kurtosis			.465												.608		
ZWATER__Ph10_Ax_3D_DISCO_C_original_firstorder_Kurtosis		-.412													.596		
ZWATER__Ph15_Ax_3D_DISCO_C_original_gldm_LargeDependenceLowGrayL		-.514														.716	

Extraction Method: Principal Component Analysis. 
 Rotation Method: Varimax with Kaiser Normalization.a	
a. Rotation converged in 15 iterations.	
Factor loadings < 0.4 were surpressed and are displayed as blank spaces. 
