# Supplementary material for: Combination of ultrafast dynamic contrast-enhanced MRI-based radiomics and artificial neural network in assessing BI-RADS 4 breast lesions: Potential to avoid unnecessary biopsies
Source: Front Oncol. 2023 Feb 1;13:1074060. doi: 10.3389/fonc.2023.1074060 (PMC9929366; doi:10.3389/fonc.2023.1074060)

**Supplemental materials 5**

**Figure S1** Diagram shows the normalized importance of each individual identified main component for DISCO-10.


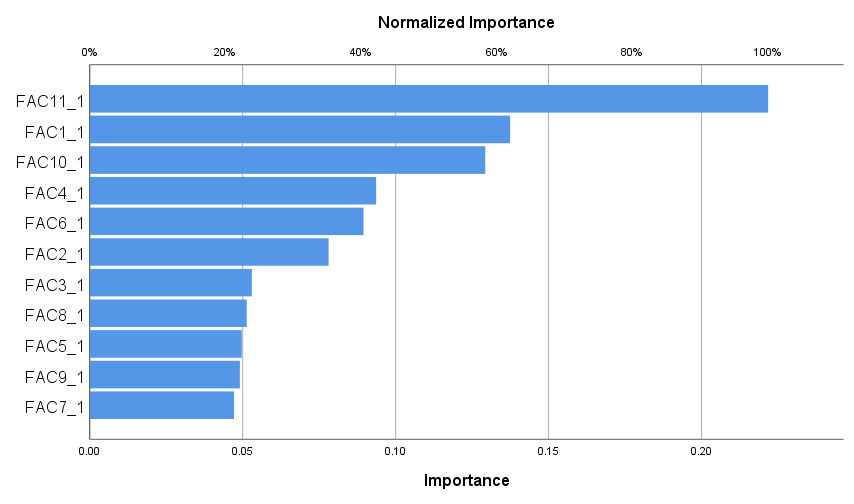


**Figure S2** ANN architecture used with one hidden layer based on the prior identified 11 main components based on DISCO-10 for predicting malignancy of breast lesions.


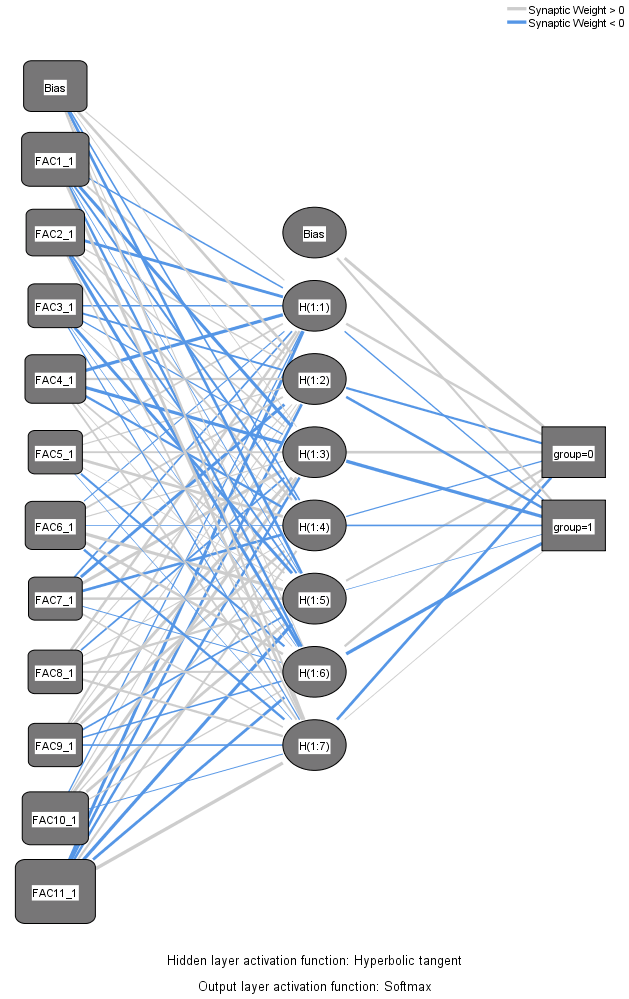


**Figure S3** Diagram shows the normalized importance of each individual identified main component for DISCO-15.


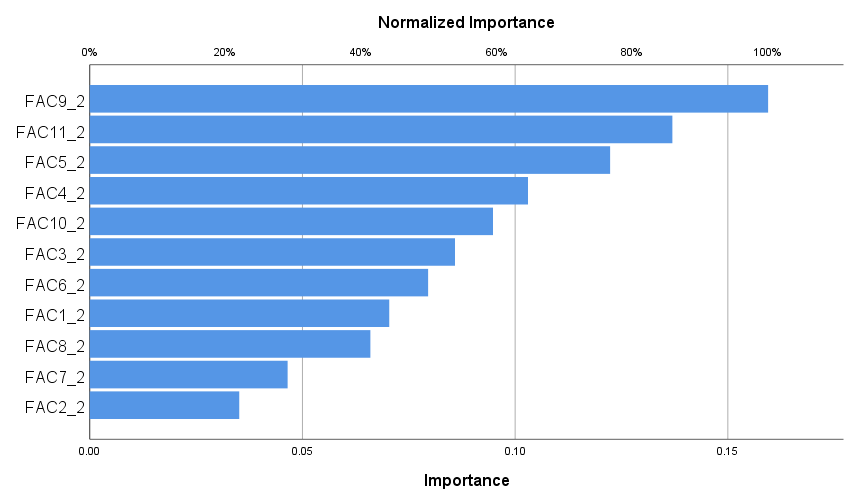


**Figure S4** ANN architecture used with one hidden layer based on the prior identified 11 main components based on DISCO-15 for predicting malignancy of breast lesions.


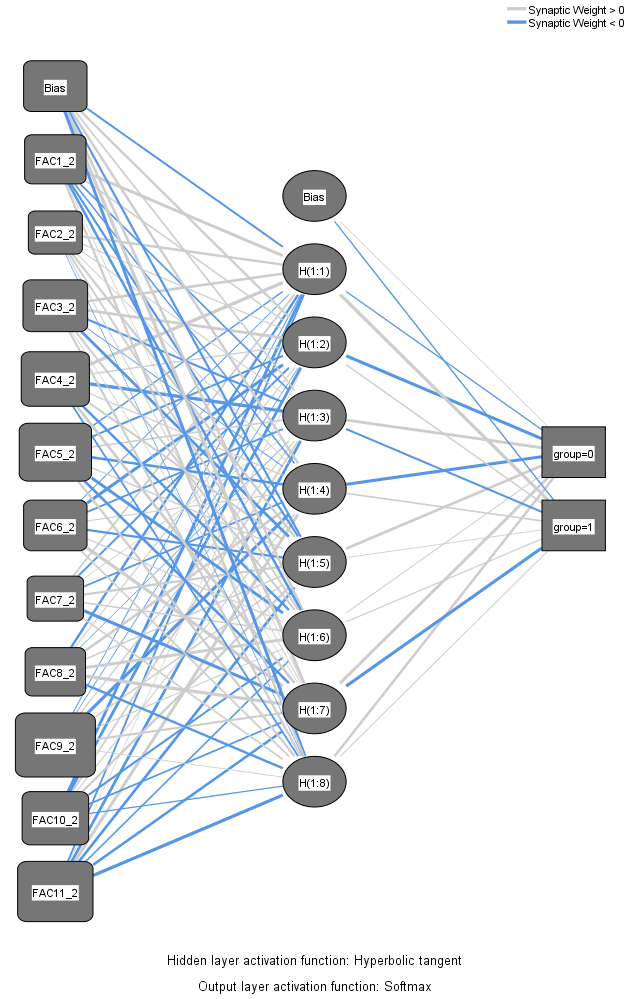


**Figure S5** Diagram shows the normalized importance of each individual identified main component for combined scheme.


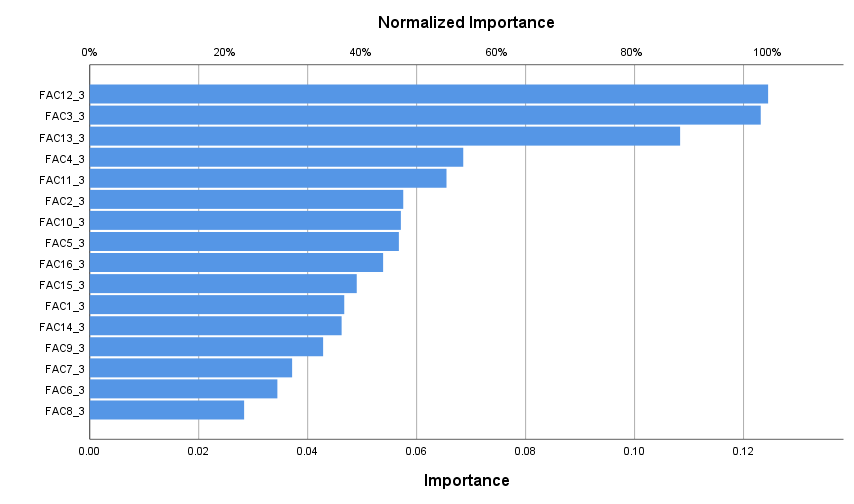


**Figure S6** ANN architecture used with one hidden layer based on the prior identified 11 main components based on combined scheme for predicting malignancy of breast lesions.


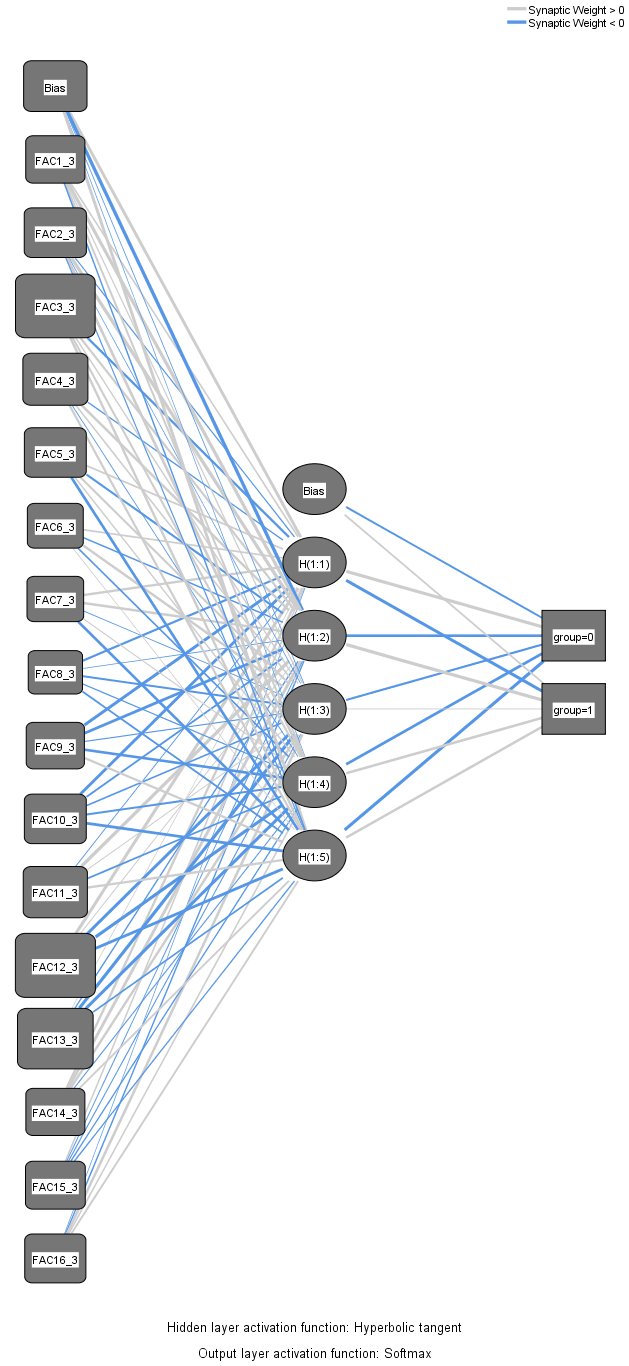

Supplement: Supplementary file 5 [file Table_5.docx]
