# Supplementary material for: Combination of ultrafast dynamic contrast-enhanced MRI-based radiomics and artificial neural network in assessing BI-RADS 4 breast lesions: Potential to avoid unnecessary biopsies
Source: Front Oncol. 2023 Feb 1;13:1074060. doi: 10.3389/fonc.2023.1074060 (PMC9929366; doi:10.3389/fonc.2023.1074060)
Supplement: Supplementary file 6 [file Table_6.docx]

**Supplementary materials 6**

**Comparison of the diagnostic performance between the radiomics models and Kaiser score**

Kaiser score (KS) is a newly proposed machine learning–derived clinical decision rule, which consists of five diagnostic features (root sign, enhancement curve type, margins, internal enhancement pattern, and edema) (1-3). The KS can provide the structure of an intuitive flowchart to guide the radiologist through a stepwise lesion assessment (1-3). The KS values include 11 rating categories ranging from 1 to 11, with each category corresponding to a distinct probability of malignancy (3). If the score exceeds 4, a biopsy is recommended. Many studies have demonstrated that the KS can improve diagnostic accuracy and has the potential to avoid unnecessary biopsies (4-7). Within the testing dataset, the difference in the AUC and specificity between ANN classifiers and the KS was not statistically significant (all *P* > 0.05) (**Tables S1-S2**). This indicated that the radiomics-based machine learning model might yield comparable results compared with the KS while not required to perform image feature interpretation.

**Table S1** Comparison for AUC of the radiomics models and Kaiser score within the testing set

|  | AUC | SE | 95%CI | *P* value* Radiomics models vs. KS |
| --- | --- | --- | --- | --- |
| DISCO-10 | 0.937 | 0.030 | 0.838 - 0.985 | 0.873 |
| DISCO-15 | 0.915 | 0.044 | 0.808 - 0.973 | 0.488 |
| Combined | 0.956 | 0.025 | 0.864 - 0.993 | 0.690 |
| KS | 0.943 | 0.030 | 0.846 - 0.988 |  |

**P* values for differences were calculated using the Delong test.

*AUC,* **area Under Curve;** *SE,* standard error; *DISCO,* Differential sub-sampling with cartesian ordering*; KS*; Kaiser score; *CI*, confidence interval

**Table S2** Comparison for specificity of the radiomics models and Kaiser score within the testing set

|  | Criterion | Sensitivity (%) (TP/TP + FN) | 95%CI | Specificity (%) (TN/TN + FP) | 95%CI | *P* value* Radiomics models vs. KS |
| --- | --- | --- | --- | --- | --- | --- |
| DISCO-10 | >0.144 | 96.00 (24/25) | 79.6-99.9 | 63.33 (19/30) | 43.9-80.1 | 0.607 |
| DISCO-15 | >0.171 | 96.00 (24/25) | 79.6-99.9 | 70.00 (21/30) | 50.6-85.3 | 1.0 |
| Combined | >0.459 | 96.00 (24/25) | 79.6-99.9 | 83.33 (25/30) | 65.3-94.4 | 0.581 |
| KS | >4 | 96.00 (24/25) | 79.6-99.9 | 73.33 (22/30) | 54.1-87.7 |  |

**P* values for differences were calculated using the McNemar test.

***TP*, true positive; *FN*, false negative; *TN*, true negative; *FP*, false positive;** *DISCO,* Differential sub-sampling with cartesian ordering*; KS*, Kaiser score; *CI*, confidence interval

1. Baltzer PA, Dietzel M, Kaiser WA. A simple and robust classification tree for differentiation between benign and malignant lesions in MR-mammography. *Eur Radiol* (2013) 23(8):2051-60. Epub 2013/04/13. doi: 10.1007/s00330-013-2804-3.

2. Marino MA, Clauser P, Woitek R, Wengert GJ, Kapetas P, Bernathova M, et al. A simple scoring system for breast MRI interpretation: does it compensate for reader experience? *Eur Radiol* (2016) 26(8):2529-37. Epub 2015/10/30. doi: 10.1007/s00330-015-4075-7.

3. Dietzel M, Baltzer PAT. How to use the Kaiser score as a clinical decision rule for diagnosis in multiparametric breast MRI: a pictorial essay. *Insights Imaging* (2018) 9(3):325-35. Epub 2018/04/05. doi: 10.1007/s13244-018-0611-8.

4. Wengert GJ, Pipan F, Almohanna J, Bickel H, Polanec S, Kapetas P, et al. Impact of the Kaiser score on clinical decision-making in BI-RADS 4 mammographic calcifications examined with breast MRI. *Eur Radiol* (2020) 30(3):1451-9. Epub 2019/12/05. doi: 10.1007/s00330-019-06444-w.

5. Milos RI, Pipan F, Kalovidouri A, Clauser P, Kapetas P, Bernathova M, et al. The Kaiser score reliably excludes malignancy in benign contrast-enhancing lesions classified as BI-RADS 4 on breast MRI high-risk screening exams. *Eur Radiol* (2020) 30(11):6052-61. Epub 2020/06/07. doi: 10.1007/s00330-020-06945-z.

6. Dietzel M, Krug B, Clauser P, Burke C, Hellmich M, Maintz D, et al. A Multicentric Comparison of Apparent Diffusion Coefficient Mapping and the Kaiser Score in the Assessment of Breast Lesions. *Invest Radiol* (2021) 56(5):274-82. Epub 2020/10/31. doi: 10.1097/RLI.0000000000000739.

7. Chen ZW, Zhao YF, Liu HR, Zhou JJ, Miao HW, Ye SX, et al. Assessment of breast lesions by the Kaiser score for differential diagnosis on MRI: the added value of ADC and machine learning modeling. *Eur Radiol* (2022). Epub 2022/06/22. doi: 10.1007/s00330-022-08899-w.
